# The Effects of a Calcium-Rich Pre-Exercise Meal on Biomarkers of Calcium Homeostasis in Competitive Female Cyclists: A Randomised Crossover Trial

**DOI:** 10.1371/journal.pone.0123302

**Published:** 2015-05-13

**Authors:** Eric C. Haakonssen, Megan L. Ross, Emma J. Knight, Louise E. Cato, Alisa Nana, Anita E. Wluka, Flavia M. Cicuttini, Bing H. Wang, David G. Jenkins, Louise M. Burke

**Affiliations:** 1 Sports Nutrition, Australian Institute of Sport, Belconnen, 2616, Australia; 2 Physiology, Australian Institute of Sport, Belconnen, 2616, Australia; 3 Performance Research, Australian Institute of Sport, Belconnen, 2616, Australia; 4 Department of Epidemiology & Preventive Medicine, School of Public Health & Preventive Medicine, Monash University, Melbourne, 3004, Australia; 5 Human Movement Studies, University of Queensland, St Lucia, 4072, Australia; 6 School of Exercise Science, Australian Catholic University, Melbourne, 3065, Australia; Tokyo Institute of Technology, JAPAN

## Abstract

Cycling is recognised as a sport in which there is a high incidence of poor bone health. Sweat calcium losses may contribute to this.

**Purpose:**

To examine whether a calcium-rich pre-exercise meal attenuates exercise-induced perturbations of bone calcium homeostasis caused by maintenance of sweat calcium losses.

**Methods:**

Using a randomized, counterbalanced crossover design, 32 well-trained female cyclists completed two 90 min cycling trials separated by 1 day. Exercise trials were preceded 2 hours by either a calcium-rich (1352 ± 53 mg calcium) dairy based meal (CAL) or a control meal (CON; 46 ± 7 mg calcium). Blood was sampled pre-trial; pre-exercise; and immediately, 40 min, 100 min and 190 min post-exercise. Blood was analysed for ionized calcium and biomarkers of bone resorption (Cross Linked C-Telopeptide of Type I Collagen (CTX-I), Cross Linked C-Telopeptide of Type II Collagen (CTX-II), Parathyroid Hormone (PTH), and bone formation (Procollagen I N-Terminal Propeptide (PINP)) using the established enzyme-linked immunosorbent assay technique.

**Results:**

PTH and CTX-I increased from pre-exercise to post-exercise in both conditions but was attenuated in CAL (p < 0.001). PTH was 1.55 [1.20, 2.01] times lower in CAL immediately post-exercise and 1.45 [1.12, 1.88] times lower at 40 min post-exercise. CTX-I was 1.40 [1.15, 1.70] times lower in CAL at immediately post-exercise, 1.30 [1.07, 1.57] times lower at 40 min post-exercise and 1.22 [1.00, 1.48] times lower at 190 min post-exercise (p < 0.05). There was no significant interaction between pre-exercise meal condition and time point for CTX-II (p = 0.732) or PINP (p = 0.819).

**Conclusion:**

This study showed that a calcium-rich pre-exercise breakfast meal containing ~1350 mg of calcium consumed ~90 min before a prolonged and high intensity bout of stationary cycling attenuates the exercise induced rise in markers of bone resorption – PTH and CTX-I.

**Trial Registration:**

Australian New Zealand Clinical Trials Registry ACTRN12614000675628

## Introduction

Prevention and treatment of low bone mineral density (BMD) is of high importance for athletes who compete in events where performance is closely related to body composition and situations of low energy availability frequently arise [1]. Cycling is recognised as a sport in which there is a high risk of poor bone health, with BMD found to be lower in cyclists compared to other athletes [2] and non-athlete controls [3]. Average reductions in BMD of ~1.5% have been reported over a cycling season [4] and increases in biomarkers of bone resorption have been detected during a three-week stage race [5]. Findings of low BMD have been observed in male adolescents [6,7] and in masters cyclists [7] as well as in professional female and male cyclists within our own lab (unpublished data). Risk factors that may underlie these phenomena include a lack of weight-bearing activity [8] as well as the low energy availability [1] that occurs due to weight loss practices and/or the high energy expenditure associated with large volumes of training and racing [4].

Recently, there has been interest in the contribution of dermal calcium losses (sweat calcium loss) during prolonged training sessions to poor bone health. Even if the athlete’s diet meets adequate intake (AI) recommendations for calcium and results in calcium balance over the day, the acute and significant dermal calcium losses during exercise may cause a decline in serum ionised calcium concentrations during exercise. Calcium has a vital role as an ion which moves in and out of the cytoplasm, acting as a signal for many cellular processes including exocytosis, neurotransmitter release, muscle contraction and the proliferation of action potentials through cardiac muscle. Due to these important functions, serum calcium concentration is defended vigorously within the body. Bone is the largest reservoir of calcium in the body and reductions in serum ionized calcium are therefore mitigated by demineralization of bone—a process stimulated by increases in parathyroid hormone (PTH). Cross linked C-telopeptide of type I collagen (CTX-I) and more recently cross linked C-telopeptide of type II collagen (CTX-II) have been indicated as sensitive markers of osteoclastic bone resorption while procollagen I N-terminal propeptide (PINP) is indicated as a marker of osteoblastic bone formation [9]. Monitoring these markers in addition to PTH can give an indication of bone metabolism in response to exercise and dietary interventions that may disrupt eucalcemia.

Daily calcium supplementation does not appear to provide a clear benefit to BMD per se [4]. However, there is some evidence that consuming a calcium supplement in close proximity to or during exercise may reduce the degree to which dermal calcium losses can impair bone health [10–12]. For example, the consumption of a ~1000 mg calcium supplement prior to [12] or during exercise [11,12] has been shown to attenuate exercise-induced increases in markers of bone resorption [12]. The supplementation protocols in these studies required the consumption of relatively high volumes of calcium fortified water; 1 L consumed 20–60 min prior to exercise or 250 ml consumed every 15 min during exercise. In practice, consuming such high volumes of fluid may not be possible or well tolerated by many athletes. The consumption of calcium-rich or fortified foods prior to exercise may confer similar benefits to bone health while also allowing athletes to meet other sports nutrition goals.

## Project Aims

The primary objective of this study was to investigate the effects of consuming calcium-rich pre-exercise foods on exercise-induced perturbations in calcium homeostasis in elite female road cyclists—a population at risk of poor bone health. It was hypothesised that a calcium-rich meal would attenuate exercise induced perturbations of bone turnover. It was also of interest to investigate any relationships between the magnitudes of exercise induced dermal calcium loss and changes in biomarkers of bone turnover with bone mineral density.

## Methods

The protocol for this trial and supporting CONSORT checklist are available as supporting information; see S1 CONSORT Checklist and S1 Text.

### Subjects

**Fig 1:**
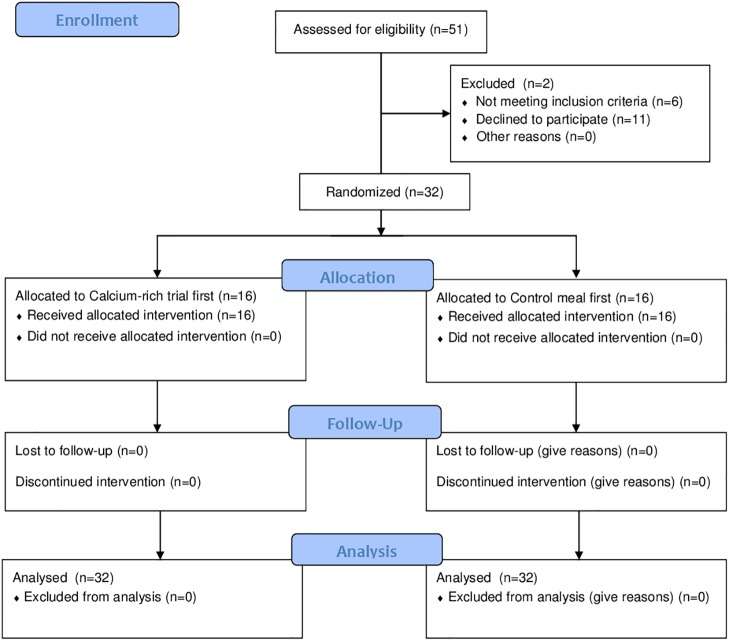
Flow chart of subject recruitment, group allocation and analysis.

Thirty-two competitive female cyclists [mean ± SD; age 24.3 ± 4.1 y, body mass (BM) 60.9 ± 7.5 kg, height 169 ± 7 cm, maximal aerobic power (MAP) 283 ± 28 W, peak oxygen consumption ($\overset{.}{\text{V}}{\text{O}}_{\text{2peak}}$) 57.1 ± 4.9 ml/kg/min] participated in the study which was held during a 10 d training camp. The Australian National Road Series (NRS) had 107 female cyclists registered at the time of this study and an average (± SD) race attendance of 47 ± 16. Fifty one cyclists expressed interested, 33 of these were NRS registered cyclists and 25 of these met the inclusion criteria (17 to 32 y; ≥18 mo racing experience; no medical condition affecting calcium homeostasis, able to commit to 10 d camp). The additional participants included an international professional, an ultra-endurance mountain biker and 5 well-trained National club-level cyclists. Exclusion criteria were vitamin D deficiency (25-OH Vitamin D < 30 ng/mL), thydroid dysfunction (n = 1), liver or kidney dysfunction, regular use of medications or supplements known to affect bone or calcium metabolism or thyroid function (e.g., thiazide diuretics, bisphosphonates, oral steroids) and allergies to cow proteins (n = 1). A flow chart illustrating subject recruitment, group allocation and analysis is shown as Fig 1. The investigation was approved by the Human Research Ethics Committee at the Australian Institute of Sport and all subjects were informed of testing protocols and risks of the study before providing signed informed consent. Subject recruitment began on 1 May 2013 and data collection commenced 13 May 2013. Subjects completed a medical questionnaire and reported having no current injury or illness. Subject follow up was completed 30 June 2013. This study was retrospectively registered as a clinical trial with the Australian New Zealand Clinical Trials Registry (ANZCTR; ACTRN12614000675628). Registration occurred after recruitment of the first subject with approval from medical advisers from the Human Research Ethics Committee at the Australian Institute of Sport. The authors confirm that all ongoing and related trials for this intervention are registered.

### Experimental design

**Fig 2:**
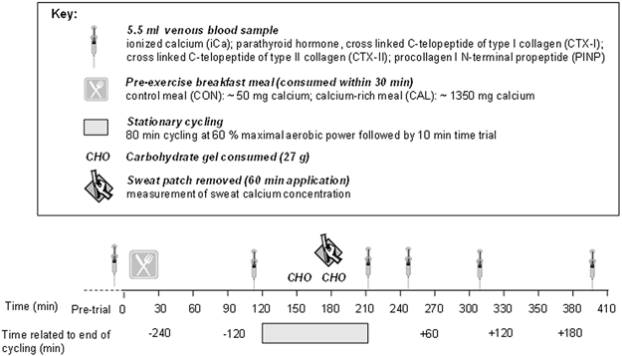
Schematic overview of study design.

All data were collected at the Australian Institute of Sport (AIS, Canberra, Australia). Cyclists performed three exercise trials in a counterbalanced cross-over order separated by one day. Maximal aerobic power output (MAP) was measured at the first visit. In a randomised order, the second and third exercise trials (60% MAP for 80 min followed by a 10 min time trial) were performed ~90 min after either a low calcium control meal or a high calcium (~1350 mg) dairy based breakfast meal. Blood was sampled on several occasions for further analysis of markers of bone turnover. An overview of the trial days is shown in Fig 2. This was a counterbalanced crossover design.

### Baseline Testing

At least 48 h prior to the first experimental trial, MAP, body composition and blood markers (8.5 ml) were assessed for exclusion criteria. Maximal aerobic power was determined using an incremental step test on an iso-power cycle ergometer (Lode Excalibur Sport, Groningen, Netherlands) starting at 125 W and increasing 25 W every 3 min until volitional fatigue. Maximal aerobic power was calculated as the highest maximum mean power output for the last 3 min of the test. Respiratory gases were analysed, as previously described [13], using a custom designed open-circuit indirect calorimetry system with associated in-house software (Australian Institute of Sport, Canberra, Australia). Bone mineral density (g/cm^2^) of the left neck of femur and lumbar spine (L_1_-L_4_) was measured by dual-energy X-ray absorptiometry (DXA; narrowed fan-beam; Lunar Prodigy; GE Healthcare, Madison, WI) with analysis performed using GE Encore 12.30 software (GE Healthcare, Madison, WI) using standardised techniques developed by Nana et al. [14].

### Experimental trials

Cyclists presented to the laboratory in a morning-fasted (~9 h) state. Following baseline blood samples (T = -15 min) athletes started the trial (T = 0 min) by consuming a pre-trial breakfast which was either a calcium-rich dairy-based meal (CAL) or low calcium control meal (CON; see *Dietary Standardisation*). Blood was sampled again immediately pre-exercise (T = 115 min); immediately post-exercise (90 min of cycle ergometry; T = 210 min); and at 40 min (T = 250 min), 100 min (T = 310 min) and 190 min (T = 400 min) post-exercise. Sweat samples were also collected after 60 min of exercise to measure sweat calcium concentration.

Subjects were randomised into one of two groups that were balanced for menstrual phase (luteal or follicular which was confirmed with measures of oestrogen and progesterone on trial day one; ovulation was avoided), menstrual regularity (amenorrhea, oligomenorrhea or regular) and use of contraceptives (oral contraceptive pills, contraceptive devices, implants or injections). Allocation concealment was not carried out. The same person who determined eligibility also determined group allocation. Group 1 received the control treatment meal first and group 2 received the treatment (calcium-rich) meal first. Subjects were sorted based on menstrual regularity (irregular or regular) and use of contraceptives. Subjects were then randomised into groups 1 and 2 using a computerised sequence generation (www.random.org) so that each group had an equal number of subjects who had a regular menstrual cycle and of those an equal number who were in their follicular or luteal phase; irregular menstrual cycle; or were using a contraceptive device. This was deemed more appropriate than using a random number generator prior to accounting for menstrual status, as menstrual status is known to effect calcium homeostasis and so balancing the groups was prioritised. Trial days began 48 h apart so that changes in the menstrual phase were minimised but allowed for sufficient recovery from exercise and time to standardise the 24 h pre-exercise diets. Both trial days started at the same time of day to avoid diurnal variation of biomarkers within subjects.

### Dietary standardisation

While housed at the AIS, subjects followed a standardised diet for 24 h prior to each experimental trial. Individualised menus were based on body weight and prepared accounting for food preferences and intolerances using FoodWorks Professional Edition (v6.0, Xyris Software, Brisbane, Australia), as previously described by Jeacocke and Burke [15]. Subjects were provided with all meals and snacks in pre-packaged form for the first 22 h of the standardised period, with the pre-trial meal prepared for subjects upon arrival at the lab 2 h prior to commencing exercise. The standardised diet was designed to provide 5.0 g/kg BM of carbohydrate (CHO); 1.5 g/kg BM of protein; 1.5g/kg BM of fat over the 24 h period. This was inclusive of 2.0 g/kg BM of CHO allocated to the pre-trial meal (see Table 1). In the case of gluten sensitivity (n = 2) and lactose-intolerance (n = 1), low lactose and gluten-free versions of foods matched for nutrient composition were used. Subjects refrained from alcohol consumption over the 24 h period but followed (and replicated) usual pre-race caffeine habits for the pre-trial meal. Compliance to the diet, determined from a self-reported checklist, was noted by a dietitian on the morning of the trial. Subjects followed standardised training on both pre-trial days (low intensity 1.5 h cycling).

**Table 1:** Macronutrient composition and calcium content of standardised diets for the control (CON) and calcium-rich (CAL) trials.

	*24 h dietary standardisation*		*Pre-exercise breakfast*	
*Nutrient*	*CON*	*CAL*	*CON*	*CAL*
	*($\overset{-}{X}$ ± SD)*	*($\overset{-}{X}$± SD)*	*($\overset{-}{X}$ ± SD)*	*($\overset{-}{X}$± SD)*
Energy (kJ/kg)	170 ± 4	169 ± 4	54 ± 2	54 ± 2
CHO (g/kg)	5.1 ± 0.2	5.1 ± 0.2	2.0 ± 0.0	2.0 ± 0.0
Protein (g/kg)	1.5 ± 0.0	1.5 ± 0.0	0.2 ± 0.0	0.6 ± 0.1
Fat (g/kg)	1.5 ± 0.0	1.5 ± 0.0	0.4 ± 0.0	0.3 ± 0.0
Calcium (mg)	640 ± 226	1658 ± 174	46 ± 7	1352 ± 53

Carbohydrate (CHO); mean ($\overset{-}{X}$); standard deviation (SD).

### Pre-exercise meal interventions

The pre-exercise meals were scaled to provide 54 kJ/kg and 2 g/kg BM CHO. The CAL meal consisted of rolled-oats cooked with calcium-fortified (Tricalcium phosphate, Nano-calcium) Anlene milk (Fonterra, Auckland, NZ), yoghurt (Yoplait, Boulogne-Billancourt, France) and additional milk, while the CON meal provided oats cooked with water and served with tinned fruit and nuts (see Table 2). The two trials differed only by the pre-trial meal, with the calcium content of the meals being 1352 ± 53 mg and 46 ± 7 mg for CAL and CON respectively. While attempts were made to ensure the two meals had a similar appearance, it was clear that one contained significantly more dairy and as such, subjects were not blinded to the meal condition.

**Table 2:** Example pre-exercise breakfast meal for control (CON) and calcium-rich (CAL) trials for a 60 kg cyclist.

*CON*		*CAL*	
Uncle Tobys Quick Oats	65 g	Uncle Tobys Quick Oats	57 g
Water	250 ml	Anlene Milk	500 ml
Goulburn Valley Fruit Salad Diced	140 g	Yoplait Yoghurt	175 g
Brown Sugar	23 g	Brown sugar	13 g
Sultana	23 g	Sultanas	20 g
Macadamias	24 g		
Meadowlea Margarine	15g		
Just Juice, Apple	200 ml		

### Exercise Protocol

Two hours after the start of the breakfast meal, cyclists began exercising on a Watttbike cycle ergometer (Nottingham, UK) at 60% of baseline MAP for 80 min (pre-load) followed by a 10 min time trial. Cyclists were instructed to maintain the highest average power possible for 10 min. The pre-load intensity of 60% MAP was chosen to be challenging enough to significantly increase sweat rate and thereby dermal losses of calcium. The time trial was used to determine whether there was any impact of pre-trial meal on cycling performance; which was previously reported by Haakonssen et al. [16]. To increase ecological validity, fans were positioned in the same location facing the athlete at a speed of 167 m/s for each trial. Room conditions were controlled at 21–22°Celsius and 50–60% relative humidity. Water was consumed *ad libitum* during the first trial and this hydration strategy was replicated during the second trial. After 30 min and 65 min of exercise, cyclists consumed a carbohydrate gel (PowerGel, PowerBar, Australia), each providing 27 g CHO.

### Blood analyses

On trial days, venous blood was collected at six time-points via a cannula: pre-trial (T = -15 min), immediately pre-exercise (T = 115 min), immediately post-exercise (T = 210 min), 40 min post-exercise (T = 250 min), 100 min post-exercise (T = 310 min) and 190 min post-exercise (T = 400 min). Blood was collected into a 2.0 mL BD Vacutainer Plus anaerobic plastic tube (Becton, Dickinson and Company, NJ, USA) to later analyse for ionized calcium (iCa), and into a 3.5 ml BD Vacutainer Plastic SST II Advance tube to measure CTX-I, CTX-II, PINP, and PTH. In trial one, at time-point one, additional blood was collected in a 3.5 ml BD Vacutainer Plastic SST II Advance tube and centrifuged for 10 min at 3000 G for measurement of progesterone and oestrogen.

Blood from the BD Vacutainer Plastic SST II Advance tube was allowed to clot by standing for 2 h at room temperature before being centrifuged at 1000 G for 10 min. Serum (0.3–0.5 ml) was then aliquoted into four 0.75 ml polypropylene tubes (Micronic America LLC, Aston, PA, USA). Blood clotting, centrifuge and freeze time were kept consistent for all samples. Once aliquoted, the samples were immediately stored at -80°C. These samples were subsequently used to measure PTH using an Enzyme-Linked immunoSorbent Assay (ELISA; IBL International GmbH, Hamburg, Germany); CTX-I using a Serum Crosslaps ELISA (Immunodiagnostics Systems Ltd. Boldon, UK); CTX-II using an ELISA (Novatein Biosciences, Woburn, USA) and PINP using an ELISA (Novatein Biosciences, Woburn, USA). Samples were batched and measured in duplicate using a SPECTROstar Nano microplate reader (BMG Labtech, Ortenberg, Germany) according to the manufacturers’ protocols. The intra-class correlation coefficients for the ELISA kits were CTX-I: r = 0.946; CTX-II: r = 0.991: PTH: r = 0.996; and PINP: r = 0.983. Sensitivities for the assays were PTH: 1.57 pg/ml; CTX-I: 0.020 ng/ml; CTX-IIL: 30.0 pg/mL; and PINP: 3.0 ng/ml. Analyses of serum were conducted at the Monash University, School of Public Health & Preventive Medicine (Melbourne, Australia).

### Adjustment for haemoconcentration

Changes in biomarkers of bone turnover were adjusted for haemoconcentration using methods described by van Beaumont et al. [17]. This method was used rather than that described by Dill and Costill [18], because haemoglobin was not measured in this study. Haematocrit (Hct) was measured using the iSTAT analyser (CG8+ cartridge, Abbott Point of Care Inc, Princeton, NJ, USA; CV 1.5% and sensitivity of 10%) and multiplied by a factor (0.96 x 0.91) to correct for plasma trapped between red blood cells and to convert venous Hct to whole-body Hct respectively. Biomarkers were then adjusted to the concentration expected (CE) based on fluid shifts alone. This was done by using the changes in Hct from immediately pre-exercise (Hct1) to all post-exercise values (Hct2) using Eq 1, where C1 represents the initial concentration of the biomarker.

CE = [Hct2(100-Hct1)/Hct1(100-Hct2] × C1 (Eq 1)

The unadjusted post-exercise biomarker concentrations (C2) were then corrected for CE using Eq 2, giving an Hct-corrected concentration (C2_Hct_).

$$\text{C}2_{\text{Hct}}=\;C2-(CE-C1)$$ (Eq 2)

All reported values for PTH, CTX-I, CTX-II and PINP were adjusted for haemoconcentration. Given that the parathyroid responds to the concentration of iCa, results for both unadjusted and adjusted iCa were reported.

### Sweat calcium analyses

The regional absorbent patch method was used to collect sweat samples during the experimental trials [19]. One patch was applied medial to the inferior angle of the right scapula for the purpose of measuring differences in sweat calcium concentration between trials. Baker and colleagues reported that when using regional skin surface sweat collection for the purpose of measuring sweat calcium concentration, no individual site nor the combination of five sites was significantly correlated with a strictly controlled whole body wash down method [20]. Therefore, only one patch was used in this study. The chosen site was cleaned with distilled water and dried with sterile gauze. An absorbent patch (Tegaderm+Pad, 3M Health Care, Minnesota, USA) was then applied 20 min prior to the start of exercise and removed 60 min into the exercise trial using aseptic techniques and placed into clean filtered centrifuge tubes (Salivette, Sarstedt AG & Co, Germany). After removal, sweat patches were immediately centrifuged at 10°C for 5 min at 3850 G to obtain a sweat sample. Sweat (0.5 mL) was added to 1 mL pooled plasma of predetermined calcium concentration in equal volumes. Sweat ionised calcium was measured on a blood gas analyser (CV <1%; Siemens RapidLab 1265, AG., Erlangen, Germany). Sweat analysis was conducted at the Cardinal Bioresearch Laboratory (New Farm, Australia).

### Data analyses

Based on results reported by Barry et al. [12] and Guillemant et al. [11], the study was sized to detect a 20 pg/mL change in the pre-post exercise serum PTH due to the administration of calcium. Calculations were performed using a one-sample T-test (two-sided), assuming a SD of the difference of 30 pg/mL, with 95% power at the 0.05 level. The effects of different pre-trial meals on Hct, iCa, PTH, CTX-I, CTX-II and PINP were investigated using linear mixed-effects modelling fit by restricted maximum likelihood estimation (REML) with pre-trial meal, time and their interaction as fixed effects and with subject nested within trial as random effects (variance component model). All response variables were log-transformed except iCa and Hct which had constant variance. Tukey’s Honestly Significant Difference (HSD) post-hoc analysis was used to identify differences. Unless stated otherwise, estimated means and 95% confidence intervals [95% CI] are reported. Differences between means and 95% CI’s for log-transformed data were back transformed to obtain estimates of the ratio of medians.

Student’s paired t-tests were used to compare total fluid loss and sweat calcium concentration between the two meal conditions. Using data from the control trial, a Spearman’s correlation matrix was created to investigate the relationship between dermal calcium loss, total sweat loss and BMD. The relationship between BMD and biomarkers of turnover (post-exercise and pre- to post-exercise change in PTH, CTX-I, CTX-II, PINP and iCa) were investigated similarly. Spearman’s correlations between biomarkers of bone turnover (PTH, CTX-I, CTX-II, PINP and iCa) were also calculated. Descriptive statistics, Z and T-Scores were calculated for BMD (g/cm^2^). Statistical analyses were conducted using JMP Pro v10 (SAS Institute Inc., Cary, NC, USA).

## Results

**Fig 3:**
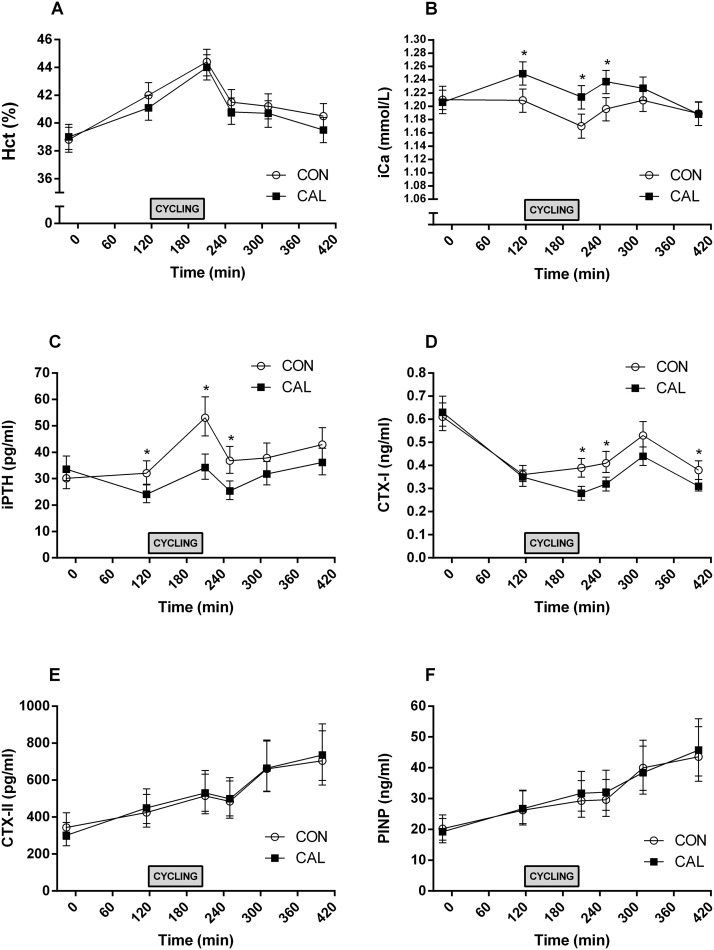
Serum concentrations of biomarkers for bone turn over, calcium homeostasis and haematocrit before and after control (CON) and calcium-rich (CAL) meal conditions and exercise. Mean ± 95% CI Haematocrit (Hct; A); ionized cacium (iCa: B); and concentrations of *parathyroid hormone* (PTH: C); *cross linked C-telopeptide of type I collagen* (CTX-I: D); *cross linked C-telopeptide of type II collagen* (CTX-II: E); *procollagen I N-terminal propeptide* (PINP: F); and at each time point for control (CON: open circles) and calcium (CAL: solid squares) trials. Blood samples were taken pre-trial at T = -15 min; pre-exercise at T = 115 min; and post-exercise at all subsequent time points. *Significant difference (p < 0.05) between trial meal conditions at the indicated time point

All 32 cyclists completed both trials. Results for Hct, iCa, PTH, CTX-I, CTX-II, and PINP are shown in Fig 3 and S2 Table. There was no interaction between *pre-trial meal* and *time* for Hct (F_5, 310_ = 1.105; p = 0.36), so this interaction was dropped from the model. There were main effects for *time* (F_5, 310_ = 75.41; p < 0.01) with Hct increasing on average 2.6 percentage units [1.8, 3.4] from T = 115 min (pre-exercise) to T = 210 min (post-exercise). There were also main effects for *pre-trial meal* (F_5, 310_ = 75.41; p < 0.01) with Hct being on average higher in CON (41.4% [40.6, 42.2]) than CAL (40.8% [40.1, 41.6]).

There was a significant interaction between *pre-trial meal* and *time* for unadjusted iCa (F_5, 310_ = 4.02; p < 0.01). Unadjusted iCa was 0.041 mmol/L higher in CAL than CON at T = 115 min [0.003, 0.078]. Unadjusted iCa significantly decreased by 0.039 mmol/L from T = 115 min to T = 210 min in CON [-0.076, -0.002]. In CAL there was a marginally significant decrease of 0.036 mmol/L [-0.072, 0.001]. At T = 210 min, unadjusted iCa was 0.044 mmol/L higher in CAL than CON [0.006, 0.082] and 0.041 mmol/L higher in CAL than CON at T = 250 min [0.004, 0.079].

There was a significant interaction between p*re-trial meal* and *time* for log(PTH) (F_5, 341_ = 6.02; p < 0.01) indicating that the effect of meal type changed depending on the time-point. Pre-Ex, the median PTH was 1.33 [1.03, 1.73] times higher in CON than the median PTH in CAL (Fig 3C). Parathyroid hormone increased significantly from T = 115 min to T = 210 min in both pre-trial meal conditions. In CAL, the median PTH at T = 210 min was 1.42 [1.10, 1.84] times higher than the median PTH at T = 115 min, while for CON the median PTH at T = 210 min was 1.66 [1.28, 2.15] times higher than the median PTH at T = 115 min. At T = 210 min, the median PTH was 1.55 [1.20, 2.01] times higher in CAL than CON. At T = 250 min, the median PTH was 1.45 [1.12, 1.88] times higher in CAL than in CON.

There was a significant interaction between *pre-trial meal* and *time* for log(CTX-I) (F_5, 310_ = 6.37; p < 0.01). The median CTX-I was estimated to be 1.40 [1.15, 1.70] times higher in CON at T = 210 min, 1.30 [1.07, 1.57] times higher at T = 250 min and 1.22 [1.00, 1.48] times higher at T = 400 min (p = 0.043) compared to CAL. At T = 310 min the median CTX-I was estimated to be 1.20 times higher (0.99, 1.45) in CON than CAL, however the lower limit of the 95% confidence interval was 0.99 suggesting that there may not have been a difference. The interaction between pre-trial meal and time was not significant for log(CTX-II) (F_5, 310_ = 0.56; p = 0.73) or log(PINP) (F_5, 310_ = 0.44; p = 0.82). There was no effect of meal type on CTX-II (F_1, 31_ = 0.02; p = 0.89) or PINP (F_1, 31_ = 0.29; p = 0.59). The only biomarkers (iCa, PTH, CTX-I, CTX-II, PINP) that were significantly correlated were CTX-II and PINP (r^2^ = 0.86; p < 0.001).

Total fluid loss was not significantly different between meal conditions (CAL 1.21 L [1.08, 1.34]; CON 1.17 L [1.04, 1.30]), with a mean difference of 0.04 L [-0.02, 0.01]. Sweat calcium concentration from the back was 10.3 mg/L [9.5, 11.2] for CAL and 10.4 mg/L [9.5, 11.2] for CON with a mean difference of 0.06 mg/L [-0.68, 0.80]. Bone mineral density was not significantly correlated with sweat calcium loss (r^2^ = 0.02; p = 0.468), total sweat loss (r^2^ = 0.05; p = 0.222) or post-exercise biomarkers of bone turnover. Bone mineral density measures are shown in Table 3. Both Z and T scores for the cyclists’ Lumbar (L_1_-L_4_) BMD were on average below zero and seven were osteopenic (T-Score <1).

**Table 3:** Bone mineral density in female cyclists.

	Neck of Femur	*Vertebrae L*_*1*_ *—L*_*4*_
	$\overset{-}{X}$ [95% CI]	$\overset{-}{X}$ [95% CI]
*BMD (g·cm* ^*-2*^ *)*	1.07 [1.11, 1.02]	1.15 [1.19, 1.10]
*Z Score*	0.29 [-0.04, 0.62]	-0.26 [-0.60, 0.00]
*T Score*	0.10 [-0.23, 0.43]	-0.41 [-0.83, 0.00]
*T-Score < -1*.*0*	n = 3 (9%)	n = 7 (22%)

Note: Bone mineral density (BMD); mean ($\overset{-}{X}$); confidence interval (CI)

## Discussion

The novel finding of this study is that the intake of a calcium-rich breakfast based on dairy sources prior to prolonged high intensity cycling attenuates the exercise-induced alterations in bone homeostasis that accompany the loss of large amounts of calcium in sweat. Specifically, a high (~1350 mg) calcium meal consumed 90 min before undertaking a 90 min bout of stationary cycling was associated with better maintained serum ionised calcium and an attenuation of the increase in markers of bone resorption (PTH and CTX-I) seen in a control trial. In contrast, markers of bone formation (PINP) were not affected by the calcium content of the pre-exercise meal. With cycling, we observed an increase in markers of resorption over ~2 h, with no change in formation markers placing the cyclist in a relatively greater resorptive state. If this occurs for several hours on most days, then bone accrual must be reduced and peak bone mass may be compromised. Elite cyclists train and compete over years in a sport with additional risk factors for poor bone mineral density [21,22]. The observations in this study suggest a practical strategy that may reduce the risk of impaired bone health frequently seen among cyclists and other endurance athletes [22].

Although it sounds counter-intuitive that athletes could suffer from poor bone health given the positive association between bone loading physical activity and bone mineral density (BMD) [8,23,24], many studies report low BMD in male and female endurance athletes arising as a direct consequence of their sports participation [22]. This has been attributed to low energy availability [1], and is further complicated by the non-weight bearing nature of sports such as cycling and swimming [22]. In cyclists where BMD values are either low [2,3,6,7] or continuing to decrease during active participation in high level cycling [4,5,25], it is of importance to identify risk factors for impaired bone health that are potentially modifiable. This has created interest in the role of increased bone resorption secondary to acute changes in serum ionic calcium concentrations arising from the dermal loss of calcium through sweat [4,11,12]. According to the limited available literature, there are indications of an increase in bone resorption during exercise involving significant sweat calcium loss, with an apparent attenuation of that negative outcome when calcium supplements are consumed before or during exercise [11,12]. In one investigation, the intake of calcium fortified (1000 mg) water prior to and during exercise was considered to reduce the potential perturbations to bone calcium, as seen by an attenuation of the significant increase in PTH associated with 60 min of cycling. In this study, the exercise-associated changes in CTX-I during and immediately after exercise were not significantly different as a result of calcium intake [12]. Meanwhile, Guillemant et al. [11] found that 30 min following completion of ~60 min of stationary cycling, the rise in CTX-I was lower as a result of calcium supplementation. Our study supports and extends these findings.

Our results provide evidence that markers of bone resorption can remain elevated for approximately 2 h following 60–90 min of cycling. Furthermore, we show that a high calcium meal sustains attenuation of the rise in CTX-I seen with exercise, with clear reductions in blood concentrations immediately and 40 min following a 90 min bout of cycling. Although CTX-II was originally proposed to be a marker of cartilage degradation [26], recent evidence suggests it is more strongly correlated with other markers of bone resorption [9] and it therefore might have been expected to mirror the movements of CTX-I. In contrast, CTX-II was not affected by the pre-exercise meal in our study, but was strongly correlated with PINP, a proposed marker of bone formation. We established a stronger relationship than has been previously reported [26], which suggests that further investigations into what aspect of bone or cartilage turnover CTX-II represents may be warranted. Differences between our results and those from previous investigations may be attributed to differences in duration and intensity of the exercise protocols previously used and differences in the time course of blood sampling. In the present study we did not sample blood during exercise; this needs to be considered when interpreting the results. However, given that Guillemant et al. [11] showed a somewhat linear increase in PTH and CTX-I during exercise it was believed that blood sampled prior to and at several time-points following exercise would be sufficient to capture exercise-induced changes in these variables and the effects of meal type.

The premise and design of our study was based on general recommendations for calcium intake for bone health as well as the specific results of two earlier investigations on calcium intake in relation to exercise [11,12]. An intake of 1000 mg of calcium per day has been indicated through meta-analysis as a threshold intake required to confer beneficial effects on BMD in combination with physical activity [27]. Similarly, 1000 mg is described as an adequate daily intake for males and females older than 19 y in the Dietary Guidelines for Australian Adults [28]. It has been suggested that a daily intake of 1500 mg would be required by male cyclists to maintain a positive calcium balance after accounting for absorption rates and sweat losses [4]. Studies utilising a calcium fortified beverage specifically around a cycling bout provided a 1000 mg calcium dose, with cyclists consuming this immediately before and/or during the exercise session [11,12]. However, this strategy requires athletes to consume 1 L of fluid, 20–60 min before exercise and 250 ml every 15 min during, which is substantially more than athletes would typically be advised to consume [29,30]. The meal utilised in this study was designed to include >1300 mg of calcium to ensure sufficient biologically-available dietary calcium in the pre-exercise meal. We found that it confers benefits to bone metabolism similar to calcium fortified water, does not impair gut comfort or time trial performance [16] and may be more practical to integrate into the athlete’s daily routine. Further work may be necessary to establish more accurately how the source, amount and timing of calcium intake interact. The necessary threshold for the described effects may be lower and may have individual variability.

Future studies should use a higher frequency of blood sampling to more accurately determine the duration at which markers of bone resorption cease to increase. Using exercise protocols with a longer duration that more accurately reflects an elite cyclist’s training load may also affect the time taken for markers of bone resorption to return to baseline. A typical training week for an elite cyclist often exceeds 20 h on the bike, with individual training sessions lasting anywhere between ~2–6 h. It is of interest to establish whether the findings from studies using comparatively short bouts of cycling (< 90 min) can be extrapolated to longer exercise periods with potentially larger dermal calcium losses. If so, this factor may well contribute to the well documented reduction of BMD reported for male and female cyclists but have a potential solution to reduce its impact. Additional work should also consider the bone turnover effects of high sweat calcium losses that occur independent of exercise or large losses that occur in conjunction with weight-bearing physical activity that is bone loading. For example, O’Toole et al. [31] showed that fire fighter recruits had no changes in BMD after 4 mo of training which included frequent bouts of high work rates lasting 3–4 h and resulting in substantial fluid losses of ~2.5 L; more than double the loss observed in the current study. It is of interest to ascertain whether weight-bearing activities are able to offset the potential effect of dermal calcium losses on bone turnover.

We also failed to find any correlations between the magnitude of exercise-induced sweat calcium losses, absolute and relative measures of biomarkers of bone turnover and BMD in the current cohort of subjects. This is in contrast to the observations of Barry and Kohrt [4] who found an inverse relationship between their estimates of dermal calcium losses and BMD in a group of male cyclists. It should be noted that these cyclists were on average 10 y older and possibly had a longer training and racing history than our subjects, potentially making a relationship between sweat calcium loss and BMD easier to identify. However, given the complex interplay between mechanical loading, nutrition and endocrine function, it could be expected that calcium sweat concentration would explain only a small proportion of the variance in BMD and indeed change in BMD over time. Clearly, long-term, prospective studies are still required to determine whether pre-exercise calcium intake (dairy or supplement) can attenuate long term reductions in BMD previously described in cyclists, particularly when other factors that contribute to bone health are difficult to alter. One known study [32] found no difference in BMD when a calcium carbonate supplement was consumed by cyclists prior to exercise for a 5 mo period. Given the subtle changes in BMD that occur over the competitive season (1.5%) [4], longer periods (> 1 y) of observation may be required.

Finally, the dairy-based pre-exercise meal used as the intervention in this study, may help athletes to achieve a number of guidelines related to health and sports nutrition goals. An increase in dairy foods in the general diet is a key recommendation of population dietary guidelines, with the recommended dietary intake (RDI) of dairy in Australia for females aged 18–60 y being 2.5 serves per day (where one serve = 1 cup milk, 200 g yoghurt or 40 g cheese) [28]. As well as the importance of dairy-derived calcium to bone health, dairy intake has been promoted, albeit controversially [33], as having a beneficial effect on body composition [34–36]. Indeed, consuming a high calcium diet in a hypoenergetic state has been shown to accelerate weight loss, with augmented effects and a greater proportion of body fat loss when dairy is the calcium source [35]. This may be important for cyclists for whom producing a relatively high power output relies on having a low body mass while maintaining sufficient functional lean mass. Guidelines for protein intake to enhance muscle protein synthesis now recommend an even spread of high quality protein sources every ~3 h over the day, and it is noted that typical food choices included in Western breakfast patterns are unlikely to meet the target of 20–25 g of protein per meal [37]. This is even more likely if dairy foods are excluded from breakfast/pre-training choices. Our dairy-rich meal provided ~36 g of protein (based on a 60 kg athlete) including a rich source of whey protein which is particularly valuable for muscle protein synthetic needs [34].

## Conclusion

While the interest in nutrition and training with elite athletes is most commonly focused directly on performance, bone health has implications for long term function and may also reduce the risk of fracture; a common outcome of accidents in road cycling races. The present study shows that a high-calcium dairy based pre-exercise meal can reduce the exercise-induced rise in markers of bone resorption. This practice can easily be incorporated into the dietary routine of endurance cyclists and may help the athlete meet other sports nutrition recommendations. Future studies should use a long-term prospective design to confirm the efficacy of pre-exercise calcium intake for improving bone health in endurance cyclists.

## Supporting Information

S1 CONSORT Checklist CONSORT Checklist. (DOCX)

S1 Text Ethics approval form. (PDF)

S2 Text Ethics application form. (PDF)

S3 Text Notification of study design amendment to AIS Ethics Committee. (PDF)

S4 Text Subject information sheet. (PDF)

S1 Table Dataset. (XLSX)

S2 Table Serum concentrations of biomarkers for bone turn over, calcium homeostasis and haematocrit before and after control (CON) and calcium-rich (CAL) meal conditions and exercise. (DOCX)
