# Supplementary material for: The Effects of a Calcium-Rich Pre-Exercise Meal on Biomarkers of Calcium Homeostasis in Competitive Female Cyclists: A Randomised Crossover Trial
Source: PLoS One. 2015 May 13;10(5):e0123302. doi: 10.1371/journal.pone.0123302 (PMC4430171; doi:10.1371/journal.pone.0123302)
Supplement: S1 Text — (PDF) [file pone.0123302.s002.pdf]

Australian Institute of Sport

MINUTE

TO:	Mr Eric Haakonssen	CC:	
FROM:	Ms Joanne Allen		
SUBJECT:	Approval from AIS Ethics Committee	DATE: 1st May 2013	

On the 9th of April 2013, the AIS Ethics Committee gave consideration to your submission titled "Effects of a high calcium pre-event meal on biomarkers of calcium homeostasis in female cyclists". The Committee saw no ethical reason why your project should not proceed subject to:

- The proposal being endorsed and approved by the Radiation Safety Committee
- The researcher following up in regards to how to handle the admission of drug or performance enhancement use by participants.
- Including a consent form for minors.
- Including the total number of hours the study will take on the Information to participants.

The approval number for this project: 20130407

It is a requirement of the AIS Ethics Committee that the Principal Researcher (you) advise all researchers involved in the study of Ethics Committee approval and any conditions of that approval. You are also required to advise the Ethics Committee immediately (via the Secretary) of:

Any proposed changes to the research design,
Any adverse events that may occur,

Researchers are required to submit **annual status reports** and **final reports** to the secretary of the AIS Ethics Committee. Details of status report requirements are contained in the "Guidelines" for ethics submissions.

Please note the approval for this submission expires on the 31st March 2014 after which time an extension will need to be sought.

If you have any questions regarding this matter, please don't hesitate to contact me on (02) 6214 1577

Sincerely
Joanne Allen
Secretary, AIS EC (Acting)
