# Supplementary material for: The Effects of a Calcium-Rich Pre-Exercise Meal on Biomarkers of Calcium Homeostasis in Competitive Female Cyclists: A Randomised Crossover Trial
Source: PLoS One. 2015 May 13;10(5):e0123302. doi: 10.1371/journal.pone.0123302 (PMC4430171; doi:10.1371/journal.pone.0123302)
Supplement: S2 Text — (PDF) [file pone.0123302.s003.pdf]

SUBMISSION TO AIS ETHICS COMMITTEE

Date of Submission: 1 May 2013

Resubmission / Version #: *(Required for minor variations and resubmissions, please include original approval number followed by version number .R1 for first revision / .R2 for second revision)*

Project Title: Effects of a high calcium pre-event meal on biomarkers of calcium homeostasis in female cyclists.

Principal Researcher	Organisation	Contact details
Mr. Eric Haakonssen	AIS - Physiology<br>Australian Sports Commission<br>PO Box 176<br>Belconnen ACT 2616	Tel: 0437013165<br>Email:<br>eric.haakonssen@ausport.gov.au

Co-Researchers	Organisation(s)
1.Prof. Louise Burke	AIS Sports Nutrition
2.Dr. David Martin	AIS - Physiology
3.Dr. Meg Ross	AIS - Physiology
4.Prof. John Hawley	RMIT - Medical Sciences
5. Assoc. Prof. Anita Wluka	Monash University - Epidemiology & Preventative Medicine
6. Prof. Flavia Cicuttini	Monash University - Musculoskeletal Unit

Brief description of the project:(this box is limited in size. ie, what you see is all the area you get)

Dairy Australia has recently formed a partnership with the Australian Institute of Sport (AIS) Sports Nutrition to undertake research and promote educational messages targeting the benefits of dairy foods in sports nutrition. The current project provides an opportunity to tackle two issues of mutual interest. The key project concerns the role of exercise-associated sweat calcium losses in the development of low bone mineral density (BMD); a major problem in both male and female cyclists. The potential for targeted intake of calcium-rich foods in the pre-exercise meal to counter this effect will be investigated by monitoring biomarkers of bone calcium homeostasis during exercise following meals of high dairy and no dairy content.

In addition, the study design also offers the opportunity to tackle a widely-held belief among athletes that dairy foods are not suitable for pre-race meals since they may cause gastric upset and general discomfort during subsequent exercise. Since the study design will compare the pre-exercise intake of a high dairy meal and the typical low dairy meal chosen by cyclists as their pre-race meal, it offers the opportunity to monitor gut comfort and cycling performance during this exercise task.

Project Aim(s):

1. To investigate the effect of a high-calcium dairy-based pre-exercise meal on exercise-associated perturbations of bone calcium homeostasis caused by sweat calcium losses

2. To compare the effects of a high dairy and low dairy pre-exercise meal on gastric tolerance and cycling performance

Background:(This box is limited in size, what you see is all the area you get, please include references at the bottom of the page)

Please include the statement of the problem, relevant literature and justification for the study.

An issue of high importance to the AIS is the prevention/treatment of low bone density in athletes: cycling is a high risk activity with low BMD found in both male and female cyclists compared with other athletes or sedentary controls, as well as a reduction in BMD over the course of a cycling season[1–3]. The range of risk factors that may underlie these phenomena include the combination of a lack of weight bearing activity, menstrual disturbances, and low energy availability due to weight loss practices or the high energy expenditure during cycling tours. An additional risk factor of interest is the acute effect of dermal calcium losses (sweat calcium loss) during prolonged training sessions. Although athletes may meet overall calcium recommended daily intakes (RDIs) and calcium balance over the day, the acute and significant dermal calcium losses during exercise may cause a decline in serum ionised calcium concentrations during exercise. Since serum calcium is vigorously defended, this may lead to an increase in serum parathyroid hormone (PTH) and a stimulation of bone re-absorption. In general, calcium supplementation does not provide a clear benefit to BMD. However, a recent study has reported that supplementation with 1000 mg of calcium prior to exercise maintains calcium homeostasis and may therefore maintain bone health[4]. This study used moderately-trained males and who ingested a calcium supplement. The proposed study will investigate whether calcium-enriched foods can attenuate exercise induced perturbations in calcium homeostasis in elite female road cyclists – a population at risk of poor bone health. It is of further interest to see if beneficial calcium could be achieved through dietary means consistent with other sports nutrition goals.

Dairy foods may not be included in the pre-race meal chosen by some road cyclists due to fears about negative 'side-effects' such as gastrointestinal discomfort or 'mucous production'. To allay such fears and to promote an increased variety of food choices in pre-event meals, there is an opportunity for research to show that a dairy-based pre-exercise meal is at least as good as other commonly consumed pre-event meals of similar carbohydrate content in the performance of endurance exercise.

Subject Information and Recruitment

Does the project involve subjects who are:

	Yes	No	N/A
AIS Scholarship holders?	☒and	☒	☐
Mentally Disabled (NS 5)?	☐	☒	☐
Physically Disabled (NS 6)?	☐	☒	☐
Minors (<18yrs) (NS 4.1, NS 4.2)?	☐	☒	☐

How are you recruiting subjects:

E-mail?	☒	☐	☐
Word of mouth?	☒	☐	☐
Referral?	☒	☐	☐
Direct correspondence with a team or coach?	☒	☐	☐
Other? Please elaborate			
Will subjects receive any monetary or other benefits for their participation (NS 1.10)?	☒	☐	☐

If **Yes** please provide further detail

Subjects will be provided with a 10 d training camp experience and all its benefits (housing, food, training education, etc.) including exposure to National Team coaching and support staff.

Description of Subjects:

Projected Number of participants: 32
Number of male: 0
Number of Female: 32
Sport/s: Cycling
Age Range: 17-35 y
Institutions involved: Cycling Australia members
Athletic Status (Elite, sub-elite, novice, recreational, sedentary): Sub-elite to elite

Criteria for participation:

Inclusion: Female, currently belonging to a state institute of sport (SIS), National Road Series (NRS) cycling team or Talent Identification (TID) program
Exclusion: Training history at this level of <18 months, Current (at the time) illness or injury, Diagnosed lactose-intolerance, Hyperparathyroidism, Vitamin D deficiency, Thyroid dysfunction, Abnormal liver or kidney function, Routine use of medications known to affect bone or calcium metabolism (e.g., thiazide diurectics, bisphosphonates, oral steroids)

Methodology: (Please use the grey text boxes)

Experimental Design: Counterbalanced crossover design with overt treatments

Detailed Methodology:

Subjects (n = 32) will be invited to participate in a 10 d AIS research and training camp. They will be provided with a copy of the plain language information prior to arrival. Before being accepted into the study subjects will be screened over the phone or in person to ensure they meet eligibility requirements and to ensure they understand what is involved. They will be housed in the AIS Residences – meals and accommodation will be provided. On day 0 (see Table 1) subjects will be addressed by the principle researcher in a briefing where logistical details of the camp and the study will be provided. Participants will be given further opportunities to ask questions (in the group setting and in private) before providing their signed informed consent to participate.

Subjects will be split into 2 groups of 16. Each subject will undergo preliminary testing and two trial days as indicated in Table 1. On the non-trial days, subjects will be invited to participate in training and a skills sessions conducted by National Team coaches.

Overview: (refer to Table 1 and Figure 1)

		GROUP 1	GROUP 2
	Subject:	1 - 16	17 - 32
Monday	13/05/2013	Pm; 2 hrs T1/T2 with paceline work	3 hrs, 4 teams (sprint Stromlo, Climb Stromlo, 3 Sisters, Sprint Stromlo, Points)
Tuesday	14/05/2013	Baseline testing Grp 1	2 hrs T1/T2 with Paceline work
Wednesday	15/05/2013	Am: 1 hr T1, Pm ; Skills (1)	Baseline testing Grp 2
Thursday	16/05/2013	Trial 1 Group 1	TTT + Black Mountain challenge
Friday	17/05/2013	Am: 1 hr T1, Pm ; Skills (2), nutrition education	4 hrs with Bridging + Skills (2)
Saturday	18/05/2013	Trial 2 Group 1	Wee Jasper ride
Sunday	19/05/2013	3 hrs, 4 teams (sprint Stromlo, Climb Stromlo, 3 Sisters, Sprint Stromlo, Points) + Skills 3	Am: 1 hr T1, Pm ; Skills (3)
Monday	20/05/2013	TTT + Black Mountain challenge	Trial 1 Group 2
Tuesday	21/05/2013	4 hrs with Bridging	Am: 1 hr T1, Pm ; Skills (1), nutrition education
Wednesday	22/05/2013	Wee Jasper ride	Trial 2 Group 2
Thursday	23/05/2013	Depart / Debrief	

Test Protocols:

Preliminary testing*:*

- Laboratory-based cycling Step test to measure aerobic capacity
- DXA measurements of body composition and BMD (according to AIS DXA research protocols, previously approved by the AIS Ethics Committee and under the guidance of the AIS Radiation Safety Officer and ARPANSA Licence

Administration)

- Anthropometry assessment (skinfolds, height, weight)
- Vitamin D status – venous blood draw (10ml)
- Dietary Restraint Questionnaire [5]

Trial Day Testing (see Figure 1)

Subjects will each undergo two trials. One trial will involve consuming a calcium enriched (CAL) dairy-based breakfast meal: 1000 mg calcium target (e.g., Flavoured Anlene milk drink + cereal with Anlene milk and high calcium yoghurt). The other trial will involve consuming a control meal (CON): Toast with jam, fruit and sports drink. These meals will be matched for carbohydrate and fluid content but not energy.

- The 24 hour diet including the pre-trial day meal will be standardised to match both trials for macronutrient and energy content. The only difference will be the trial day breakfast calcium content.
- **Pre-Trial:** Subjects will present to the lab fasted where they will be cannulated and a baseline blood will be drawn (10mm)
- Depending on the treatment order, subjects will be provided with either the CAL or CON breakfast meal described above
- **Pre-Load Exercise:** Two hours following their first mouthful, subjects will complete 80 min steady state pre-load cycling on the Lode ergometer at ~60% VO_2max
- **Performance Measure:** Following the steady state cycling cyclists will complete a 10 min time trial on the Velotron ergometer
- During exercise, heart rate (HR) and rate of perceived exertion (RPE) will be recorded every 10 min.
- Subjects will be provided with 3 x 41g carbohydrate Powerbar gels, with one consumed at the start of exercise, at 30 min and after 60 min exercise. Water will be consumed *Ad Libitum*
- **Blood samples:** 8ml blood samples will be collected via cannula at four times, as indicated in Figure 1. These will be assayed for serum Parathyroid Hormone (PTH), serum ionized calcium (iCA), N-terminal propeptides (PIN P), C-terminal telopeptide of type I and Type II collagen (CTX)
- **Sweat collection:** sweat will be collected using an established patch technique. This involves placing a filter paper disc (~42 mm) on the skin under a Parafilm dressing. Patches are placed on chest, scapula, thigh and both forearms.
- The patches will be collected every 30 min during exercise and analysed for calcium concentration.
- Following completion of the 30 min time trial subjects will be given 30 min to get changed and report to the kitchen where they will be provided with a post-exercise meal. They will remain there until 3 h post-exercise at which point the final blood will be collected and the cannula removed.

Questionnaires:

- **Preliminary Testing:** Health and training history questionnaire (history of bone and menstrual health, family history bone health, history of Vitamin D status, training history)
- **During trials:** Gastrointestinal comfort questionnaire (five-point Likert scale; Gut Q

– see below) will be administered every 30min during exercise and for 1.5 h post-exercise (see Figure 1)

- **Palatability Questionnaire (VAS):** to determine whether both meals are equally palatable
- **Appetite Questionnaire (VAS):** to determine whether both meal provide the same level of satiety [6]

Figure 1. Trial Timeline

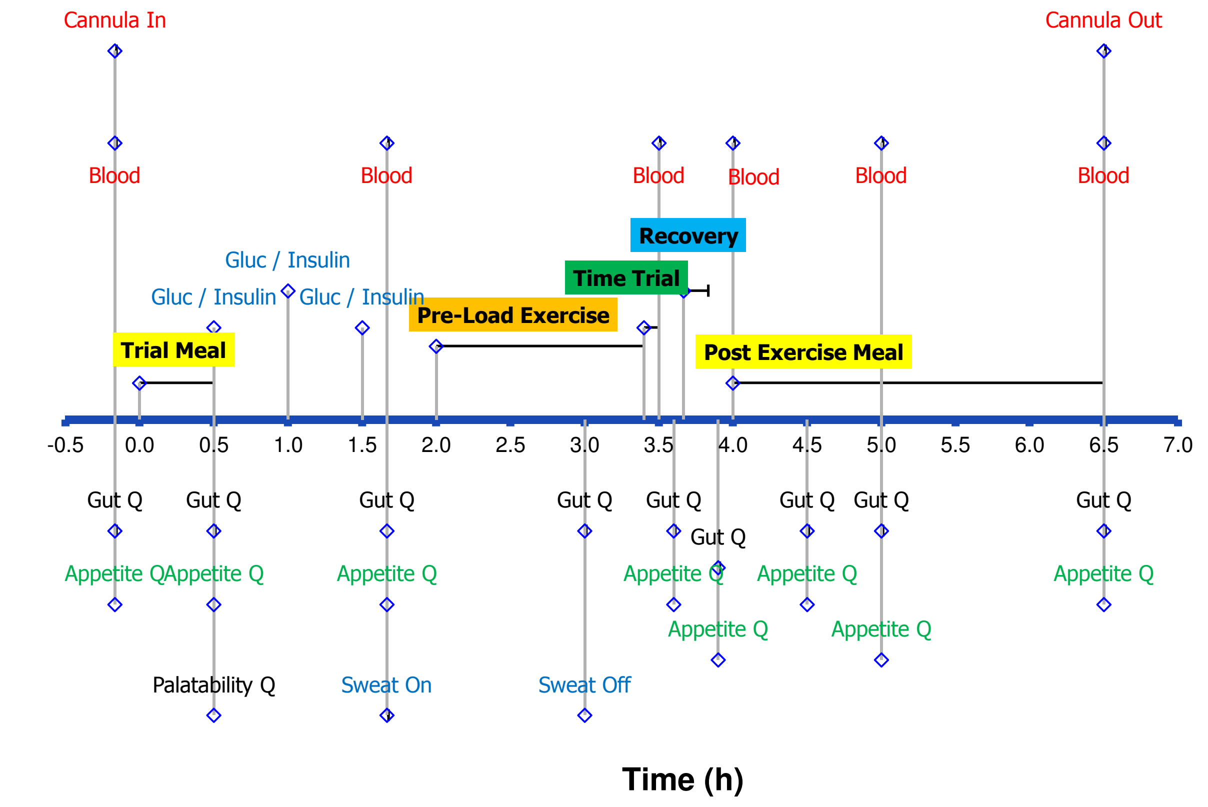

Data Analysis:

Outcome variables

- Plasma glucose concentration following pre-event meal and during exercise
- Gut comfort during and after exercise
- Bone turnover and calcium homeostasis (PTH, iCA, PINP, CTX)
- Sweat loss, sweat calcium loss
- Performance (10 min time trial; time (min) and average power output (W))

Normal statistical treatments will be undertaken on the data:

- Repeated Measures ANOVA will be used to investigate changes and differences in biological parameters.
- Correlation analyses will investigate relationships between current BMD and sweat calcium losses or indices of perturbed calcium homeostasis due to exercise. Power calculations based on Barry et al., (2011) suggest that at least 20 subjects are required for sufficient power with markers of bone turnover and calcium

homeostasis[4]

- Magnitude based inferences (using Will Hopkins' spreadsheets) will be used to investigate performance differences

Ethical Considerations:

Biomedical Procedures:

	Yes	No	N/A
Does this proposal involve Biomedical Procedures (NS 14, NS 15)?	☒	☐	☐

If **YES** explain the procedures:
DXA measurements of body composition and bone mineral density (according to AIS DXA research protocols, previously approved by the AIS Ethics Committee and under the guidance of the AIS Radiation Safety Officer and ARPANSA Licence Administration)

Subjects will be cannulated to allow blood sampling at multiple time points:

- Pre-meal (10 ml: bone/calcium markers PTH, iCA, PINP, CTX)
- Pre-exercise, post-exercise, and 3 h post-exercise (10 ml: bone/calcium markers)

In addition, capillary blood samples will be taken at various intervals to measure blood glucose

- 30, 60 and 90 min post-meal
- During exercise: 30, 60 and 90 min

	Yes	No
Has a qualified medical practitioner approved the procedures:	☐	☒

If **NO** explain why:
Biomedical procedures will be carried out by appropriately qualified AIS Physiology staff who have met laboratory standards. This is a routine procedure previously performed in the AIS Physiology lab.

*Please ensure medical officer signs the bottom of this application.

Radiation Exposure:

	Yes	No
Will this study involve drugs or chemical agents, ionising radiation, non-ionising radiation or high intensity sound *(Including DEXA)* (NS 10)?	☒	☐
If **YES**, have you sought advice from the external Radiation Safety Committee?	☐	☒

This will be completed by Helene Rushby, AIS Performance Research.

Blinding:

	Yes	No	N/A
Does this proposal involve procedures specifically designed to directly modify the knowledge, thinking, attitudes, feelings or other aspects of the behaviour of subjects (NS 17.1 NS 17.2)?	☐	☒	☐
If **YES**, does this study involve giving false/misleading information to subjects or withholding information such that their "informed" consent is in question (NS 17.1, NS 17.2)?	☐	☒	

Procedural:

	Yes	No	N/A
Are the procedures new or innovative (not established) (NS 13)?	☐	☒	☐
Will the procedures cause any degree of discomfort, harassment, invasion of privacy, risk of physical injury, threat to dignity of subjects or be otherwise potentially harmful to subjects (NS 1.3)?	☒	☐	☐

Explain:

Please provide further detail: During the cycling performance measures we will ask that subjects exercise to maximal exertion, which will likely cause some discomfort. However, this will be a usual sensation experienced by cyclists of this calibre. Cycling training conducted on the open road will carry an increased risk of accident or injury. However, this risk will be minimised by escorting the group with a sign-posted vehicle driven by experienced personnel who are appropriately trained/accredited first-aiders. There may be also some perceived discomfort experienced by subjects during anthropometric measurements and the DXA scan, as this requires minimal clothing to be worn. Again, these measurements will be carried out by experienced personnel in a professional manner in order to maximise decency and reduce the time required to be partially undressed.

Security and Anonymity:

Describe how you will maintain the anonymity of participants (NS 1.19, NS 15.9, NS 18)? Hard-copies of recording sheets and printed raw-data will be stored in a lockable filling cabinet located at the AIS. Electronic data will be stored on the Australian Sports Commission (ASC) server that is password protected and accessible only to AIS physiology members. Subjects' identity will be decoded by replacing names with subject ID (number) and this information will be held by the Principle investigator. All data will be made available only to research staff directly associated with this project. A final report of the pooled data will not contain any text that identifies individuals.

What will be gained by undertaking the research?

Explain how the benefits outweigh the risks (NS 1.14)? The findings of this study could provide dietary recommendations that could attenuate the decline in bone health in this population. This has implications for long term health in female cyclists as well as other endurance athletes. Furthermore, the subjects will be given the opportunity receive training, education and recovery from National Team coaches as well as exposure to these coaches who will be recruiting subjects for an end of year selection camp. The cost of accommodation and meals will be covered and all training rides will be well supported.

Project Details:

Proposed time-frame (NS 1.16):	Month / Year
Design:	April / 2013
Ethics:	April / 2013
Recruitment:	April / 2013
Commencement:	May / 2013
Data Analysis:	July / 2013
Report:	November / 2013

Absolute completion: December / 2013

Give estimates for:

Total average time required for subject's participation (in hours)- ~20 h testing including explanations and preparation

The total number of items if questionnaires/tests are involved- 2 questionnaires and 3 laboratory-based testing days

(Please note that Ethics Approval is only valid for 3 months post proposed written completion date, notification to the Committee via the Secretary will be required for an extension to the time frame)

Budget required: **$ 100,000**

Approved level of funding: **$ 100,000**

Source(s) of funding: **Dairy Australia Grant**

How will the results of the study be implemented or used? (eg what impact will the results have on the daily training environment)

The findings from the study will be presented to Cycling Australia coaches and athletes via newsletter and will also be shared in person with Dieticians and other Sports Science staff working within Cycling Australia.

How will the results of the study be presented? (eg written report, published papers, thesis, conference, seminars)

The findings from the study will be submitted for publication in an international peer-reviewed scientific journal and PhD thesis. This work will be presented at international sports science conferences and for university candidature requirements.

'INFORMED CONSENT' FORM (Adult)

Project Title: **Effects of a high calcium pre-event meal on biomarkers of calcium homeostasis in female cyclists**

Principal Researchers: Eric Haakonssen, Prof. Louise Burke, Dr. Meg Ross

This is to certify that I, ______________________ hereby agree to participate as a volunteer in a scientific investigation as an authorised part of the research program of the Australian Sports Commission under the supervision of _________________.

The investigation and my part in the investigation have been defined and fully explained to me by ___________________ and I understand the explanation. A copy of the procedures of this investigation and a description of any risks and discomforts has been provided to me and has been discussed in detail with me.

- I have been given an opportunity to ask whatever questions I may have had and all such questions and inquiries have been answered to my satisfaction.
- I understand that I am free to deny any answers to specific items or questions in interviews or questionnaires.
- I understand that I am free to withdraw consent and to discontinue participation in the project or activity at any time, without disadvantage to myself.
- I understand that I am free to withdraw my data from analysis without disadvantage to myself.
- I understand that any data or answers to questions will remain confidential with regard to my identity.
- I certify to the best of my knowledge and belief, I have no physical or mental illness or weakness that would increase the risk to me of participating in this investigation.
- I am participating in this project of my (his/her) own free will and I have not been coerced in any way to participate.

Signature of Subject: ______________________________ Date: ___/___/___

I, the undersigned, was present when the study was explained to the subject/s in detail and to the best of my knowledge and belief it was understood.

Signature of Researcher: ____________________________ Date: ___/___/___

Please note NS refers to the NHMRC 'National Statement' for the ethical conduct of research. For further information please go to http://www.nhmrc.gov.au and follow the links

‘INFORMED CONSENT’ FORM (Minor)

Project Title: **Effects of a high calcium pre-event meal on biomarkers of calcium homeostasis in female cyclists**

Principal Researchers: Eric Haakonssen, Prof. Louise Burke, Dr. Meg Ross

This is to certify that I, hereby agree to give permission to have my child participate as a volunteer in a scientific investigation as an authorised part of the research program of the Australian Sports Commission under the supervision of .

The investigation and my child’s part in the investigation have been defined and fully explained to me by and I understand the explanation. A copy of the procedures of this investigation and a description of any risks and discomforts has been provided to me and has been discussed in detail with me.

- I have been given an opportunity to ask whatever questions my child or myself may have had and all such questions and inquiries have been answered to my satisfaction.
- I understand that my child is free to deny any answers to specific items or questions in interviews or questionnaires.
- I understand that my child is free to withdraw consent and to discontinue participation in the project or activity at any time, without disadvantage.
- I understand that my child is free to withdraw his/her data from analysis without disadvantage.
- I understand that any data or answers to questions will remain confidential with regard to my child’s identity.
- I certify to the best of my knowledge and belief, my child has no physical or mental illness or weakness that would increase the risk to me (him/her) of participating in this investigation.
- My child is participating in this project of my (his/her) own free will and My child has) not been coerced in any way to participate.

Signature of Participant: _______________________________ Date: ___/___/___

Signature of Parent or
Guardian of minor: (under 18 years) ______________________ Date: ___/___/___

I, the undersigned, was present when the study was explained to the subject/s in detail and to the best of my knowledge and belief it was understood.

Signature of Researcher: _____________________________ Date: ___/___/___

Please note NS refers to the NHMRC ‘National Statement’ for the ethical conduct of research. For further information please go to http://www.nhmrc.gov.au and follow the links

INFORMATION PRIVACY PRINCIPLES IN PLAIN ENGLISH

Principle 1 - Restricting collection of information to lawful purposes and by fair means.
Agencies must not collect personal information unless (i) it is collected for a lawful purpose directly related to a function the agency; and (ii) the means of' collection are lawful and fair.

Principle 2 - Informing people why information is collected.
Agencies must ensure that people from whom they solicit personal information are generally aware, before collection, or as soon as practical thereafter, of (i) the purpose of collection; (ii) any legal authority for the collection; and (iii) any third parties to which the collecting agency discloses such information as a usual practice.

Principle 3 - Ensuring personal information collected is of good quality and not too intrusive.
Where an agency solicits personal information (whether from the person that the information is about or otherwise), it must take reasonable steps to ensure (i) that the information is relevant to the purpose of collection, up-to-date and complete; and (ii) that its collection does not unreasonably intrude upon the person's personal affairs.

Principle 4 - Ensuring proper security of personal information
An agency must protect personal information against misuse by reasonable security safeguards, including doing everything within its power to ensure that authorised recipients of the information do not misuse it.

Principle 5 - Allowing people to know what personal information is collected and why.
Any person has a right to know whether an agency holds any personal information (whether on him or her or not), and if so (a) its nature; (b) the main purposes for which it is used; (c) the classes of persons about whom it is kept; (d) the period for which each type of record is kept; (e) the persons who are entitled to have access to it, and under what conditions; and (f) how to obtain access to it. Each agency must maintain a register of this information and must inform the Privacy Commissioner annually of its contents.

Principle 6 - Allowing people access to their own records.
A person has a right of access to personal information held by an agency, subject to exceptions provided in the Freedom of Information Act 1982 or any other law.

Principle 7 - Ensuring that personal information stored is of good quality, including allowing people to obtain corrections where it is not.
Agencies must make corrections, deletions and additions to personal information to ensure that it is (i) accurate; and (ii) relevant, up-to-date, complete and not misleading (given the purpose of collection and related purposes), subject to exceptions provided in the Freedom of Information Act 1992 or any other law. Agencies are also required to add a reasonable statement by a person to that person's record, on request.

Principle 8 - Ensuring that personal information is of good quality before using it. Agencies must take reasonable steps to ensure that personal information is accurate, up-to-date and complete (given the purpose of collection and related purposes) before using it.

Principle 9 - Ensuring that Personal information is relevant before using it.
Agencies may only use personal information for purposes to which it is relevant.

Principle 10 - Limiting the use of personal information to the purposes for which it was collected.
Agencies may not use personal information for purposes other than for which it was collected except (a) with the consent of the person; (b) to prevent a serious and imminent threat to a person's life or health; (c) as required or authorised by law; (d) where reasonably necessary for the enforcement of criminal or revenue laws; or (e) for a directly related purpose in the case of exception (d), but not otherwise, the use must be logged.

Principle 11 - Preventing the disclosure of personal information outside the agency.
Agencies may not disclose to anyone else personal information, with the same exceptions as apply to Principle 10 (a) - (d), plus an additional exception where the subject of the information is reasonably likely to be aware of the practice of disclosure (or reasonably likely to have been made aware under Principle 2). The recipient of information under one of these exceptions may only use it for the purpose for which it was disclosed.

I, Eric Haakonssen acknowledge the Information Privacy Principles and undertake to ensure that these principles are adhered to with reference to collection of data from subjects, for the purpose of this research project.

Signed……………. .... Principal Researcher

Date …...1/5/2013...

FINAL CHECK LIST:

	Yes	No	N/A
Are procedures for obtaining consent fully described in a copy of the "informed consent" form (NS 1.7, 1.8, 1.12, 4.1, 4.2, 5 and 6.7)	☒	☐	☐
Have you provided a copy of the ***Information to Participants***?	☒	☐	☐
Have you completed and provided a copy of the ***Informed Consent***?	☒	☐	☐
Has the proposal been endorsed by the appropriate AIS Head Coach?	☒	☐	☐
Has the proposal been endorsed by an appropriate **medical officer?**	☐	☒	☐
Has funding been approved for the project?	☒	☐	☐
Has the appropriate Head of Discipline* read and endorsed the proposal? (Proof is required – email will suffice)	☒	☐	☐
Have you read, understood and signed the '***Privacy Principles***' agreement?	☒	☐	☐

*Applicants external to the AIS must forward their proposal to the appropriate AIS Head of Department

Signed: ………………………………………………………………… / /

Principal Researcher Date

AIS Head of Department Endorsement

I acknowledge that I have thoroughly read the submission and am satisfied that the methodology has sufficient rigour and that the research team has the appropriate resources and expertise to perform the study.

Print name: ………Professor Louise Burke………

Sign: Louise Burke ……………………………………….. 1 / 5 / 13

Note: Where the Principal Researcher is Head of Discipline, then their supervisor should provide the above endorsement.

Please note NS refers to the NHMRC 'National Statement' for the ethical conduct of research. For further information please go to http://www.nhmrc.gov.au and follow the links

Medical Officer Endorsement

I acknowledge that I have thoroughly read the submission and am satisfied that the biomedical procedures meet the appropriate medical stands, have sufficient rigour and that the research team has the appropriate resources and expertise to perform the study.

Print name: ……………………………………………………………

Sign: ………………………………………..   /   /

.

Appetite Questionnaire

Subject____________________ Date____________

Condition: *calcium / control* *CWI / Stretch*

Time administered (staff to circle one)

1) *Before pre-trial meal*
2) *Post pre-trial meal*
3) *Pre-Exercise*
4) *Post Time Trial*
5) *Post Recovery*
6) *Post Exercise Meal*
7) *End of Trial*

Instructions

With your pen, mark along the line to indicate how much you feel the response to the left or right of the line reflects your own response to the question in the middle.

	**How hungry do you feel?**	
I am not hungry at all	______________________________	I have never been more hungry
	**How satisfied do you feel?**	
I am completely empty	______________________________	I cannot eat another bite
	**How full do you feel?**	
Not at all full	______________________________	Totally full
	**How much do you think you can eat?**	
Nothing at all	______________________________	A lot
	**Would you like to eat something sweet?**	
Yes, very much	______________________________	No, not at all
	**Would you like to eat something salty?**	
Yes, very much	______________________________	No, not at all
	**Would you like to eat something savoury?**	
Yes, very much	______________________________	No, not at all
	**Would you like to eat something fatty?**	
Yes, very much	______________________________	No, not at all

Palatability Questionnaire

Subject____________________ Date____________

Administered after pre-trial meal: *calcium* / *control*

Instructions

With your pen, mark along the line to indicate how much you feel the response to the left or right of the line reflects your own response to the question in the middle.

Good	**Visual appeal**	Bad
Good	**Smell**	Bed
Good	**Taste**	Bad
Much	**Aftertaste**	None
Good	**Palatability**	Bad

Subject____________________ Date____________

Condition: *calcium* / *control*

How comfortable does your stomach feel at the moment?

1	Very Comfortable
2	Comfortable
3	Average Comfort
4	Uncomfortable
5	Very Uncomfortable

**Time Point (post meal)**	**Response**
2.0 hr	
2.5 hr	
3.0 hr	
3.5 hr	
4.0 hr	
4.5 hr	
5.0 hr	
5.5 hr	
6.0 hr	
6.5 hr	

Dietary Restraint Questionnaire – Baseline Testing

Subject____________________ Date____________

Part 1

Please read each statement carefully and indicate your response by circling T (true) or F (false).

1.	When I smell a sizzling steak or see a juicy piece of meat, I find it very difficult to keep from eating, even if I have just finished a meal.	T	F
2.	I usually eat too much at social occasions, like parties and picnics.	T	F
3.	I am usually so hungry that I eat more than three times a day.	T	F
4.	When I have eaten my quota of calories, I am usually good about not eating any more.	T	F
5.	Dieting is so hard for me because I just get too hungry.	T	F
6.	I deliberately take small helpings as a means of controlling my weight.	T	F
7.	Sometimes things just taste so good that I keep on eating even when I am no longer hungry.	T	F
8.	Since I am often hungry, I sometimes wish that while I am eating, an expert would tell me that I have had enough or that I can have something more to eat.	T	F
9.	When I feel anxious, I find myself eating.	T	F
10.	Life is too short to worry about dieting.	T	F
11.	Since my weight goes up and down, I have gone on reducing diets more than Once.	T	F
12.	I often feel so hungry that I just have to eat something.	T	F
13.	When I am with someone who is overeating, I usually overeat too.	T	F
14.	I have a pretty good idea of the number of calories in common food.	T	F
15.	Sometimes when I start eating, I just can't seem to stop.	T	F
16.	It is not difficult for me to leave something on my plate.	T	F
17.	At certain times of the day, I get hungry because I have gotten used to eating then.	T	F
18.	While on a diet, if I eat food that is not allowed, I consciously eat less for a period of time to make up for it.	T	F
19.	Being with someone who is eating often makes me hungry enough to eat also.	T	F
20.	When I feel blue, I often overeat.	T	F
21.	I enjoy eating too much to spoil it by counting calories or watching my weight.	T	F
22.	When I see a real delicacy, I often get so hungry that I have to eat right away.	T	F
23.	I often stop eating when I am not really full as a conscious means of limiting the amount that I eat.	T	F
24.	I get so hungry that my stomach often seems like a bottomless pit.	T	F

25.	My weight has hardly changed at all in the last 12 mths years.	T	F
26.	I am always hungry so it is hard for me to stop eating before I finish the food on my plate.	T	F
27.	When I feel lonely, I console myself by eating.	T	F
28.	I consciously hold back at meals in order not to gain weight.	T	F
29.	I sometimes get very hungry late in the evening or at night.	T	F
30.	I eat anything I want, any time I want.	T	F
31.	Without even thinking about it, I take a long time to eat.	T	F
32.	I count calories as a conscious means of controlling my weight.	T	F
33.	I do not eat some foods because they make me fat.	T	F
34.	I am always hungry enough to eat at any time.	T	F
35.	I pay a great deal of attention to changes in my figure.	T	F
36.	While on a diet, if I eat a food that is not allowed, I often then splurge and eat other high calorie foods.	T	F
37.	If I eat a little bit more on one day, I make up for it the next day.	T	F
38.	I pay attention to my figure, but I still enjoy a variety of foods.	T	F
39.	I prefer light foods that are not fattening.	T	F
40.	If I eat a little bit more during one meal, I make up for it at the next meal.	T	F
41.	Do you deliberately restrict your intake during meals even though you would like to eat more?	T	F
42.	I eat diet foods, even if they do not taste very good.	T	F
43.	A diet would be too boring a way for me to lose weight.	T	F
44.	I would rather skip a meal than stop eating in the middle of one.	T	F
45.	I alternate between times when I diet strictly and times when I don't pay much attention to what and how much I eat.	T	F
46.	Sometimes I skip meals to avoid gaining weight.	T	F
47.	I avoid some foods on principle even though I like them.	T	F
48.	I try to stick to a plan when I lose weight.	T	F
49.	Without a diet plan I wouldn't know how to control my weight.	T	F
50.	Quick success is most important for me during a diet.	T	F

Part 2

Please answer the following questions by circling the number above the response that is appropriate to you.

1. How often are you dieting in a conscious effort to control your weight?

1	2	3	4
Rarely	Sometimes	Usually	Always

2. Would a weight fluctuation of 2 kg affect the way you live your life?

1	2	3	4
Not at all	Slightly	Moderately	Very much

3. How often do you feel hungry?

1	2	3	4
Only at meal times	Sometimes between meals	Often between meals	Almost always

4. Do your feelings of guilt about overeating help you to control your food intake?

1	2	3	4
Never	Rarely	Often	Always

5. How difficult would it be for you to stop eating halfway through dinner and not eat for the next four hours?

1	2	3	4
Easy	Slightly difficult	Moderately difficult	Very difficult

6. How conscious are you of what you are eating?

1	2	3	4
Not at all	Slightly	Moderately	Extremely

7. How frequently do you avoid ‘stocking up’ on tempting foods?

1	2	3	4
Almost never	Seldom	Usually	Almost always

8. How likely are you to shop for low calorie foods?

1	2	3	4
Unlikely	Slightly unlikely	Moderately likely	Very likely

9. Do you eat sensibly in front of others and splurge alone?

1	2	3	4
Never	Rarely	Often	Always

10. How likely are you to consciously eat slowly in order to cut down on how much YOU eat?

1	2	3	4
Unlikely	Slightly unlikely	Moderately likely	Very likely

11. How frequently do you skip dessert because you are no longer hungry?

1	2	3	4
Almost never	Seldom	At least once a week	Almost everyday

12. How likely are you to consciously eat less than you want?

1	2	3	4
Unlikely	Slightly unlikely	Moderately likely	Very likely

13. Do you go on eating binges though you are not hungry?

1	2	3	4
Never	Rarely	Sometimes	At least once a week

14. On a scale of 0 to 5, where 0 means no restraint in eating (eating whatever you want, whenever you want it) and 5 means total restraint (constantly limiting food intake and never ‘giving in’), what number would you give yourself?

0 Eat whatever you want, whenever you want it

1 Usually eat whatever you want, whenever you want it

2 Often eat whatever you want, whenever you want it
3 Often limit food intake, but often "give in"
4 Usually limit food intake, rarely "give in"
5 Constantly limiting food intake, never "giving in"

15. To what extent does this statement describe your eating behaviour? 'I start dieting in the morning, but because of any number of things that happen during the day, by evening I have given up and eat what I want, promising myself to start dieting again tomorrow.'

1	2	3	4
Not like me	Little like me	Pretty good description of me	Describes me perfectly

Part 3

Please answer the following questions by circling the number above the response that is appropriate to you.

1. In the last 14 days, have you dieted in a conscious effort to control your weight?

1	2	3	4
Not at all	Slightly	Moderately	Very much

2. Do you intend to diet during this camp in a conscious effort to control your weight?

1	2	3	4
Not at all	Slightly	Moderately	Very much

3 In the last 14 days, have you used any of the following techniques for the specific purpose of reducing body weight? (check ☒ as many as apply)

- ☐ I reduced food intake throughout the day
- ☐ I avoided foods high in fat
- ☐ I increased daily training duration
- ☐ I avoided foods high in sugar
- ☐ I wore additional clothes or plastic wraps when training
- ☐ I skipped breakfast, lunch or dinner
- ☐ I performed long training rides (>2hrs) without eating
- ☐ I avoided eating after training
- ☐ I used weight loss supplements or medications
- ☐ I made myself vomit after eating
- ☐ Describe others:
- ☐ None of the above

____

____

____
