# Supplementary material for: The Effects of a Calcium-Rich Pre-Exercise Meal on Biomarkers of Calcium Homeostasis in Competitive Female Cyclists: A Randomised Crossover Trial
Source: PLoS One. 2015 May 13;10(5):e0123302. doi: 10.1371/journal.pone.0123302 (PMC4430171; doi:10.1371/journal.pone.0123302)

1st May, 2013

Joanne Allen
Secretary, AIS EC
Australian Institute of Sport

Dear Joanne,

**Re: Ethics application amendment for project**

We would like to advise all members of the AIS Ethics Committee of the proposed amendments to the research design of the project entitled "*Effects of a high calcium pre-event meal on biomarkers of calcium homeostasis in female cyclists*", as required.

Briefly, the study design changes that we would like the committee to consider are listed as follows:

1. **The addition of an Appetite and Palatability Questionnaire.** In order to determine whether the two pre-trial meals are equally palatable and provide a similar degree of satiety, we would like the subjects to complete a visual analogue scale following the trial meals and throughout the day as indicated in timeline below. This scale has previously been validated (Flint et al., 2000).
2. **The addition of a Dietary Restraint Questionnaire.** This questionnaire would be administered at the initial camp brief and will help the investigators characterise the subjects and determine whether they are prone to manipulating their diets and whether they intend to do so during the study. This questionnaire has been validated (Westenhoefer et al., 1999). Should there be any adverse findings; AIS Nutrition will provide additional counselling to the subjects.
3. **The addition of one trial day blood draw.** To increase the resolution of biomarkers we are testing for we would like to take six instead of five blood samples during the trial day. The blood tests will now require only an 8 ml draw instead of the originally proposed 10 ml which results in the same volume of blood drawn and because these are coming from a cannula there will be no additional needles.
4. **The removal of capillary blood samples for glucose.** we will no longer require the use of a capillary blood sample to measure blood glucose as originally proposed given that we have found an iStat cartridge that allows us to run blood glucose from the same sample used for other analytes.
5. **Pre-trial day dietary standardisation.** We would like to standardise the pre-trial day meals from lunch time the day before. This will ensure that the 24 hr macronutrient composition and energy content for both trials are the same and only the calcium content of the pre-trial breakfasts differ. The meals will provide the athletes with 5g CHO/kg over the 24 hr period and will be based on foods in the attached list. They will be contacted prior to the trials to ensure we know their food preferences and we do not give them anything they dislike or are allergic to.

6. **Change of biomarkers measure.** Following advice from our collaborators at Monash University we have decided not to measure bonealkaline phosphatase (BAP) and instead measure N terminal propeptides (PIN P) as it is a more reliable marker of bone metabolism. This will have no additional burden on subjects.

We would like to thank the committee members in advance for their consideration and apologise for this unforseen inconvenience. Please find attached copies of the updated AIS Ethics Application and Plain Language Statement. We look forward to hearing about the outcome of our amended application before proceeding further with this project.

Kind regards,

Eric Haakonssen
Principle Investigator
AIS Physiology

## References


Flint, A., Raben, A., Blundell, J.E., Astrup, A., 2000. Reproducibility, power and validity of visual analogue scales in assessment of appetite sensations in single test meal studies. Int J Obes Relat Metab Disord 24, 38.

Westenhoefer, J., Stunkard, A.J., Pudel, V., 1999. Validation of the flexible and rigid control dimensions of dietary restraint. International Journal of Eating Disorders 26, 53–64.


**Figure 1. Trial Timeline**

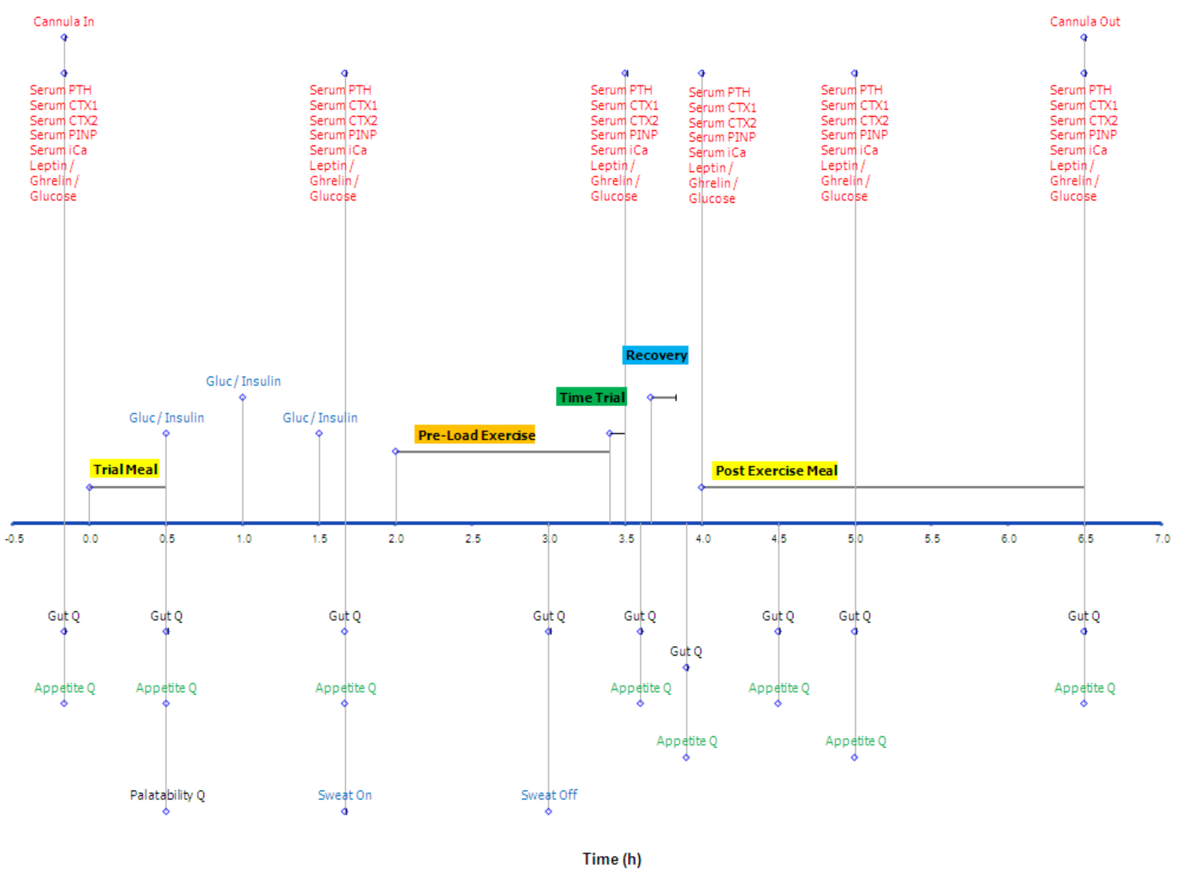

Supplement: S3 Text — (PDF) [file pone.0123302.s004.pdf]
