# Supplementary material for: The Effects of a Calcium-Rich Pre-Exercise Meal on Biomarkers of Calcium Homeostasis in Competitive Female Cyclists: A Randomised Crossover Trial
Source: PLoS One. 2015 May 13;10(5):e0123302. doi: 10.1371/journal.pone.0123302 (PMC4430171; doi:10.1371/journal.pone.0123302)
Supplement: S4 Text — (PDF) [file pone.0123302.s005.pdf]

AUSTRALIAN INSTITUTE OF SPORT

Australian Government
Australian Sports Commission

Information for Participants

Effects of a high-calcium pre-event meal on biomarkers of calcium homeostasis in female cyclists

Mr. Eric Haakonssen; Prof. Louise Burke; .Dr. Meg Ross; Prof. John Hawley; Ms. Anita Wluka; Ms. Flavia Cicuttini; Dr. David Martin

- Thank you for your interest in participating in a research study, which is being conducted in conjunction with a training camp supported by the Australian Institute of Sport (AIS) – Sports Nutrition program.

- This research project and all procedures have been approved by the AIS human ethics committee. Project 20130407.

- Your participation is **completely voluntary** and you may discontinue your participation at any time.

- **Background:** An issue of high importance to the AIS is the prevention/treatment of low bone density in athletes: low bone mineral density (BMD) is common in both male and female cyclists. This may be because of a lack of weight-bearing activity, menstrual disturbances, and low energy availability due to weight loss practices or the high energy expenditure. An additional risk factor of interest is the acute effect of sweat calcium loss. These sweat calcium losses during prolonged exercise may cause a significant decline in blood calcium concentrations during training. The body protects blood calcium levels by stimulating the bones to release calcium into the blood to restore levels, and increasing bone re-absorption.

- **Purpose:** to determine whether a high calcium pre-event meal can reduce levels of hormones that are responsible for increasing bone re-absorption.

- This study will be run in conjunction with Cycling Australia as part of a **ten day training-camp** held at the AIS. Each cyclist who volunteers will be housed at the AIS in Canberra for the duration of the camp and all meals and snacks will be provided. There will be a total **of three testing days** – one preliminary testing day and two trial days.

- **Preliminary Testing:** On the preliminary testing day we will collect data to describe your physical and endurance characteristics. You will be asked to complete a health and training history questionnaire which will ask you about the health and regularity of your menstrual cycle. You will also complete a maximal aerobic capacity step test where you ride a stationary cycle ergometer to exhaustion while breathing into a mouthpiece that collects the air you expire (VO_2max test). You will have a blood test to determine your Vitamin D status. You will also have your body composition measured as described below – anthropometry and DXA. Finally we will ask you to complete a Dietary Restraint Questionnaire while help us understand how much you purposefully alter your diet.

- **Anthropometry:** Your height (cm) weight (kg) and skin folds (mm) will be measured. Skin fold measurements will be taken from 7 sites on your body. This minimally invasive assessment will be performed by an accredited Anthropometrist who will gently pinch and measure the thickness of the skin fold (the skin and underlying adipose tissue) on the front and back of your arm, your back, the front of your hips, stomach, thigh and calf. This procedure will take ~15 min to complete and will be

performed in a private room staffed by the tester and a recorder. Please note that you will be required to wear minimal clothing (e.g., sports bra and knicks) in order to complete this test.

- **Dual-energy X-ray absorptiometry (DXA)** is a technology most commonly used to provide information on bone density in research and clinical situations. However, whole body scans using DXA technology can be used to estimate total body fat and lean body mass. They can also provide assessment of body composition (volume of lean muscle, bone and adipose tisues) in specific body regions – legs, arms and torso. This information may be useful to monitor changes due to targeted training programs or during rehabilitation from injury. Before accepting you into the study we will ask you to undertake a brief questionnaire that provides information about your training and sporting background, and identifies a couple of conditions that will exclude you from having a DXA scan and thus, participating in the study. These exclusion criteria are:
    - Being pregnant or breastfeeding
    - Being unable or uncomfortable with lying still on the DXA bed while the scan is performed
    - Having had recent or planned exposure to radiation such as normal X-ray, or other medical tests such as CT scan

Participation in the study will require you to come to the Physique and Fuel Centre at the AIS. On the test day, you will need to come in each morning under standard conditions:

- **Overnight fasted.** This means you must not eat anything and can drink only water on the morning before your test
- **Rested.** You should not exercise on the morning before you arrive at the Centre. Your last exercise session should have finished before 8 pm on the previous evening
- **Well hydrated.** We will ask you to drink a certain amount of fluid on the evening before the test and will get you to collect a sample of your “first- waking” urine so that we can check your hydration levels

We are all exposed to sources of radiation in everyday life, including radiation in the surrounding environment. This study will require you to undertake three DXA scans (one body composition scan and two bone mineral density scans) during one visit, which delivers a very small dose of radiation (total of ~1.9 µSv). To put this radiation dose into perspective, it is less radiation than the amount that is received during a 7 hour aeroplane flight (~50 µSv) and much less than the typical radiation exposure with conventional x-rays (25 -60µSv).

If you have further concerns about this small level of radiation, we have had a medical physicist prepare an assessment of the radiation doses that we will use in this study. This report is available to read on request.

The estimated total dose from this study is less than 200 µSv and the risk assessment for radiation effects from a dose assessment report carried out by Mr. David Leslie (Manager, Compliance Testing and Consultancy, Gammasonics Radiological Services) is less than 10^{-5}.

Testing Trials

There will be two trial days which are illustrated below in Figure 1. In each trial you will be required to give six blood samples. In order to do this we will insert a cannula into your arm so that you only have one needle. The cannula will remain in your arm for the duration of the test, which will allow us to simply ‘turn on’ and ‘turn off’ a tap for multiple blood collections. One trial will involve consuming a calcium enriched (CAL) dairy-based breakfast meal: 1000 mg calcium target (e.g., Flavoured Anlene milk drink + cereal with Anlene milk and high calcium yoghurt). The other trial will involve consuming a control meal (CON): Toast with jam, fruit and sports drink. Two hours later you will complete 80 min of

moderate-intensity exercise on a stationary bicycle followed by a maximal 10 min time trial. During the tests the researchers will collect very small (8ml) samples of blood from the cannula and sweat from sweat patches (small absorbent pad stuck to your skin with a clear 5 x 7 cm adhesive film) placed on your forearms, chest and shoulder. We will also ask you to complete questionnaires of appetite before and after the trial meals and exercise as well as a gastrointestinal comfort questionnaire. We will ask you to complete a palatability questionnaire after the meals to see if you like the way they taste. After the time trial you will be provided with a meal, but we will ask you to remain in the laboratory for 3 hours after the trial so we can collect the final blood sample at which time the cannula will be removed.

Figure 1. Trial Timeline

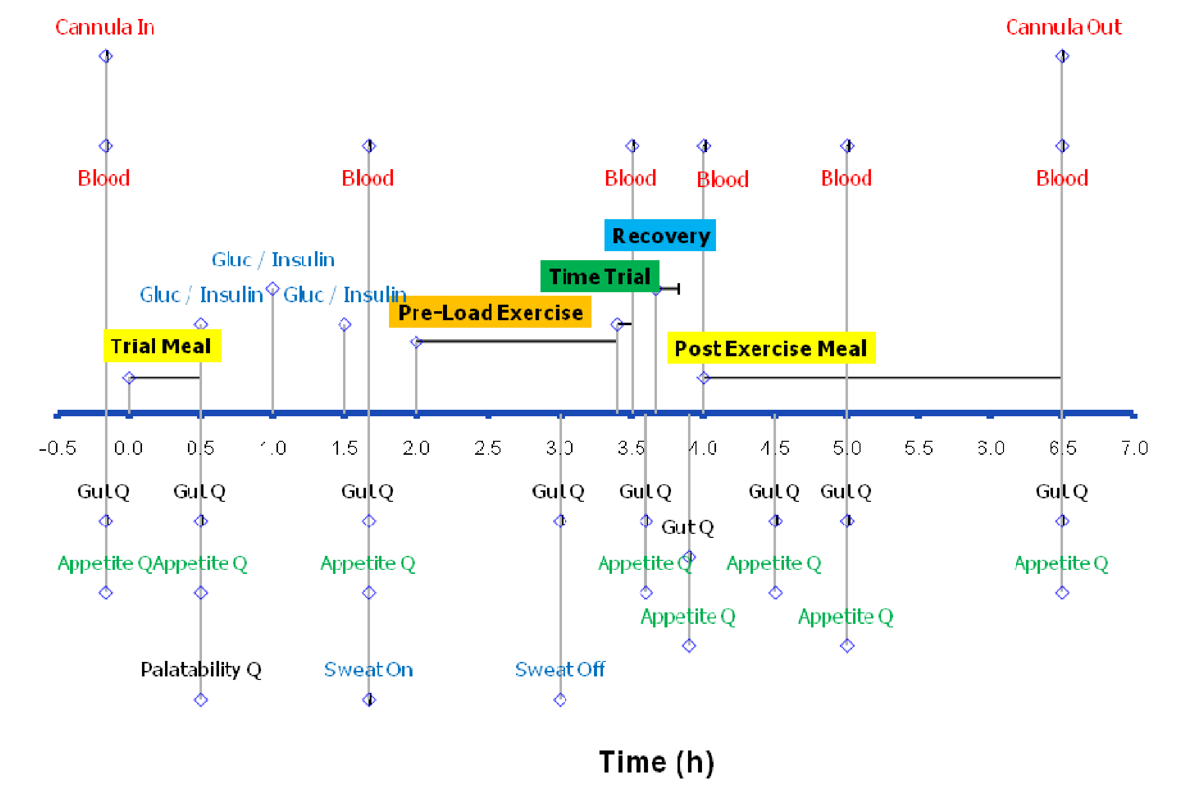

Trial Day Time Requirement

The trial day will last for almost 7 hrs which will include meals and time where you will be required to remain under supervision until. There will be 30 min allocated to the trial meal, 1.5 hr waiting until the start of the exercise component of the trial. The exercise will last for 1.5 hr. The post exercise meal will begin 30 min later and you will then have 2.5 hr until the final blood is drawn where you will be asked to remain under supervision.

Benefits to you

In return for your time during the camp and effort in testing, you will be involved in other aspects of a training camp run by Cycling Australia coaches (National Team Head Coach – Martin Barras) and supported by AIS Physiology and Sports Nutrition programs. This will involve fully-supported training rides, skills sessions, access to recovery facilities and a range of educational material as well as reports on your performance data. In addition, all meals (including snacks and training food) and accommodation will be provided at no cost to you.

Your rights

It is important to us that subjects participate in this study without incident, accident, illness or injury, and we hope that you will discuss with us any concerns or queries that would prevent you from completing the study. You are free to withdraw at any time, without any pressure or repercussions.

All the information collected from you will be treated with strict regard to your privacy and confidentiality. You will be identified by number only, and all contact details and information will be kept securely in the primary investigator's care and only be available for the small group of investigators involved in this study (listed above). The information that is collected from you will be made available to you, along with the **group** results (mean data) from all subjects. Your results will not be released to anyone else without your written permission, and you will not be individually identified in any written reports or presentations. We may take photographs of testing sessions for the purpose of presentations, however, your permission will be sought before these are taken.

Should we find any health results that are of concern, you will be notified and given advice regarding the appropriate health professional to consult.

- **All data will be blinded** by team and age and each athlete will get a reference copy of their data which includes the percentiles for the measurements.

- Your personal information will be kept **confidential** and will be made available only to principle researchers on this study. Should you wish, your cycling performance measures will be shared with AIS / Cycling Australia coaching staff and sports scientists.

- Should you have any questions regarding your involvement, you may contact myself or Helene Rushby, Sports Research Consultant and AIS Ethics Committee Secretary (02 6214 1577) or helene.rushby@ausport.gov.au

Thank you for your time.

Eric Haakonssen
Physiology
Australian Institute of Sport
Australian Sports Commission
Tel: +61 2 6214 7887
Mob: +61 449887603
eric.haakonssen@ausport.gov.au
