# Supplementary material for: The Effects of a Calcium-Rich Pre-Exercise Meal on Biomarkers of Calcium Homeostasis in Competitive Female Cyclists: A Randomised Crossover Trial
Source: PLoS One. 2015 May 13;10(5):e0123302. doi: 10.1371/journal.pone.0123302 (PMC4430171; doi:10.1371/journal.pone.0123302)
Supplement: S2 Table — (DOCX) [file pone.0123302.s007.docx]

**S7 Table**

| **Serum concentrations of biomarkers for bone turn over, calcium homeostasis and haematocrit before and after control (CON) and calcium-rich (CAL) meal conditions and exercise** | | | | | |
| --- | --- | --- | --- | --- | --- |
| *Biomarker* | *Time* | *CON* | | *CAL* | |
|  | *(min)* | $\bar{X}$ [95% CI] | | $\bar{X}$ [95% CI] | |
| Hct (%) | -15 | 38.8 | [37.9, 39.7] | 39.0 | [38.1, 39.9] |
|  | 115 | 42.0 | [41.1, 43.0] | 41.1 | [40.2, 42.0] |
|  | 210 | 44.4 | [43.4, 45.3] | 44.0 | [43.1, 44.9] |
|  | 250 | 41.5 | [40.5, 42.4] | 40.8 | [39.9, 41.8] |
|  | 310 | 41.2 | [40.3, 42.1] | 40.7 | [39.7, 41.6] |
|  | 400 | 40.5 | [39.6, 41.4] | 39.5 | [38.6, 40.4] |
| Unadjusted  iCa (pg/ml) | -15 | 1.21 | [1.19, 1.23] | 1.21 | [1.19, 1.22] |
|  | 115 | 1.21 | [1.19, 1.23] | 1.25 | [1.23, 1.27]* |
|  | 210 | 1.17 | [1.15, 1.19] | 1.21 | [1.20, 1.23]* |
|  | 250 | 1.20 | [1.18, 1.21] | 1.24 | [1.22, 1.25]* |
|  | 310 | 1.21 | [1.19, 1.23] | 1.23 | [1.21, 1.24] |
|  | 400 | 1.19 | [1.17, 1.21] | 1.19 | [1.17, 1.21] |
| iCa (pg/ml) | -15 | 1.21 | [1.18, 1.25] | 1.21 | [1.17, 1.24] |
|  | 115 | 1.21 | [1.18, 1.24] | 1.25 | [1.22, 1.28] |
|  | 210 | 1.10 | [1.07, 1.13] | 1.13 | [1.09, 1.16] |
|  | 250 | 1.21 | [1.18, 1.24] | 1.25 | [1.21, 1.28] |
|  | 310 | 1.23 | [1.20, 1.26] | 1.24 | [1.21, 1.27] |
|  | 400 | 1.23 | [1.20, 1.27] | 1.20 | [1.17, 1.23] |
| PTH (pg/ml) | -15 | 30.11 | [26.20, 34.60] | 33.60 | [29.24, 38.60] |
|  | 115 | 32.03 | [27.87, 36.80] | 24.04 | [20.92, 27.63]* |
|  | 210 | 53.05 | [46.16, 60.96] | 34.19 | [29.75, 39.28]* |
|  | 250 | 36.75 | [31.99, 42.23] | 25.37 | [22.08, 29.16]* |
|  | 310 | 37.85 | [32.94, 43.50] | 31.74 | [27.62, 36.47] |
|  | 400 | 42.93 | [37.36, 49.33] | 36.11 | [31.43, 41.49] |
| CTX-I (ng/ml) | -15 | 0.61 | [0.55, 0.67] | 0.63 | [0.57, 0.70] |
|  | 115 | 0.36 | [0.33, 0.40] | 0.35 | [0.31, 0.38] |
|  | 210 | 0.39 | [0.35, 0.43] | 0.28 | [0.25, 0.31]* |
|  | 250 | 0.41 | [0.37, 0.46] | 0.32 | [0.29, 0.35]* |
|  | 310 | 0.53 | [0.48, 0.59] | 0.44 | [0.40, 0.49] |
|  | 400 | 0.38 | [0.34, 0.42] | 0.31 | [0.28, 0.34]* |
| CTX-II (pg/ml) | -15 | 343.94 | [279.70, 422.94] | 300.91 | [244.70, 370.02] |
|  | 115 | 424.39 | [345.13, 521.86] | 449.77 | [365.77, 553.07] |
|  | 210 | 514.24 | [418.19, 632.35] | 530.18 | [431.16, 651.95] |
|  | 250 | 484.47 | [393.98, 595.74] | 499.05 | [405.84, 613.67] |
|  | 310 | 660.03 | [536.75, 811.62] | 664.38 | [540.29, 816.97] |
|  | 400 | 704.72 | [573.09, 866.57] | 735.40 | [598.05, 904.31] |
| PINP (ng/ml) | -15 | 20.21 | [16.52, 24.73] | 18.21 | [15.70, 23.50] |
|  | 115 | 26.20 | [21.42, 32.50] | 26.74 | [21.86, 32.71] |
|  | 210 | 29.29 | [23.94, 35.83] | 31.73 | [25.94, 38.82] |
|  | 250 | 29.62 | [24.22, 36.24] | 32.07 | [26.22, 39.24] |
|  | 310 | 39.97 | [32.67, 48.90] | 38.43 | [31.41, 47.01] |
|  | 400 | 43.60 | [35.64, 53.35] | 45.70 | [37.35, 55.91] |
| Note: Haematocrit (Hct); Ionized calcium (iCa); parathyroid hormone (PTH); cross linked C-telopeptide of type I collagen (CTX-I); cross linked C-telopeptide of type II collagen (CTX-II); procollagen I N-terminal propeptide (PINP); mean ($\bar{X}$); confidence interval (CI).  *Significant difference (p < 0.05) | | | | | |
